# Supplementary material for: Scanless two-photon voltage imaging
Source: Nat Commun. 2024 Jun 14;15:5095. doi: 10.1038/s41467-024-49192-2 (PMC11178882; doi:10.1038/s41467-024-49192-2)
Supplement: Supplementary file 1 — Supplementary Information [file 41467_2024_49192_MOESM1_ESM.pdf]

Scanless two-photon voltage imaging

Supplementary Information

Ruth R. Sims[1]*, Imane Bendifallah[1]*, Christiane Grimm[1], Aysha S. Mohamed Lafirdeen[1], Soledad Domínguez[1], Chung Yuen Chan[1], Xiaoyu Lu[2], Benoît C. Forget[1], François St-Pierre[2,3,4,5], Eirini Papagiakoumou[1§], and Valentina Emiliani[1§]

[1]Institut de la Vision, Sorbonne Université, INSERM, CNRS, F-75012 Paris, France

[2]Systems, Synthetic, and Physical Biology Program, Rice University, Houston, TX, USA

[3]Department of Neuroscience, Baylor College of Medicine, Houston, TX, USA

[4]Department of Biochemistry and Molecular Pharmacology, Baylor College of Medicine, Houston, TX, USA

[5]Department of Electrical and Computer Engineering, Rice University, Houston, TX, USA

*1st author equal contribution

§valentina.emiliani@inserm.fr

§eirini.papagiakoumou@inserm.fr

This file includes:

- Supplementary Notes 1 - 2
- Supplementary Tables 1 - 6
- Supplementary Methods
- Supplementary Figures 1 - 18
- Supplementary References

Table of contents

Supplementary Tables – Experimental setups used for scanless two-photon voltage imaging

1. Summary of optical components used for two-photon (2P) voltage imaging
2. Summary of optical components used for 2P voltage imaging with low repetition rate laser @ 1030 nm
3. Summary of optical components used for in vivo 2P voltage imaging
4. Experimental configurations
5. Experimental protocols
6. Comparative table of 2P optical approaches

Supplementary Methods

1. Extracting fluorescence time series from scanless 2P voltage imaging data
2. Calibration procedures

Supplementary Figures

1. Optical setup for scanless 2P voltage imaging
2. Summary and evaluation of data analysis pipeline
3. Comparison of different parallel illumination approaches for scanless 2P voltage imaging (protocol 1)
4. Comparison of different parallel illumination approaches for scanless 2P voltage imaging (protocol 2)
5. Comparison of different parallel illumination approaches for scanless 2P voltage imaging (protocol 3)
6. Comparison of scanless 2P voltage imaging between isolated CHO cells and densely labelled hippocampal organotypic slices
7. Characterisation of scanless 2P voltage imaging for imaging trains of action potentials and subthreshold activity
8. Quantification of perturbations induced by scanless 2P voltage imaging upon illumination with laser pulses at high repetition rate.
9. Evaluation of temperature rise in tissue upon scanless voltage imaging with high repetition rate laser pulses
10. 940 vs 1030-nm illumination comparison, evaluation of sensitivity curve for low repetition rate illumination
11. Evaluation of temperature rise in tissue upon scanless voltage imaging with low repetition rate laser pulses in vitro
12. Quantification of perturbations induced by scanless 2P voltage imaging upon illumination with laser pulses at low repetition rate.
13. Multi-target scanless 2P voltage imaging with low repetition rate illumination at 1030 nm
14. In vivo scanless 2P voltage imaging
15. Evaluation of temperature rise in tissue upon scanless voltage imaging with low repetition rate laser pulses in vivo
16. All-optical in situ characterization of photo evoked action potentials
17. Evaluation of temperature rise in tissue upon scanless voltage imaging with low duty cycles and single-beam photoactivation and voltage imaging

Supplementary Tables

Supplementary Table 1: List of optical components of the main setup used for 2P voltage imaging on organotypic slices (Setup 1)

Component	Description	Manufacturer, Part Reference
Laser A	Tuneable femtosecond source, tuned to 920, 940 or 1030 nm (1.4 W, 80 MHz, 100 fs)	Coherent, Chameleon Discovery
Laser B	Femtosecond source, fixed output 920 nm (4W, 80 MHz, 100 fs)	Alcor, Spark Lasers
Laser C	Custom OPA pumped by amplified laser, fixed output 940 nm (0.5 W, 250 kHz, 100 fs)	Amplitude, Satsuma Niji
Laser D	Femtosecond source, fixed output 1030 nm (40 MHz, max power 5 W, 150 fs)	Amplitude, Goji
λ/2	Half-wave plate	Thorlabs, WPHSM05-980
PBS	Polarizing beam splitter	Thorlabs, CCM1-PBS253/M
MS	Mechanical Shutter or high-speed modulator	Thorlabs, SH05R/M or OM6NH/M
L1	Lens, focal length = 80 mm	Thorlabs, AC508-80-B
L2	Lens, focal length = 300 mm (GPC) or 200 mm (Low-NA Gaussian beam)	Thorlabs, AC508-300-B or AC508-200-B
SLM1	Spatial Light Modulator, 600 x 800 pixels, 20 μm pitch	Hamamatsu, LCOS 10468-07
L3	Lens, focal length = 400 mm	Thorlabs, AC508-400-B
PCF	Phase Contrast Filter, 60 μm radius	Double Helix Optics, custom design
L4	Lens, focal length = 300 mm	Thorlabs, AC508-300-B
G1	Blazed diffraction grating, 600 lines/mm	Richardson Gratings
L5	Lens, focal length = 500 mm	Thorlabs, AC508-500-B
SLM2	Spatial Light Modulator, 600 x 800 pixels, 20 μm pitch	Hamamatsu, LCOS 10468-07
L6	Lens, focal length = 500 mm	Thorlabs, AC508-500-B
L7	Lens, focal length = 300 mm	Thorlabs, AC508-300-B
L8	Lens, focal length = -75 mm	Thorlabs, LC1258-B
L9	Lens, focal length = 500 mm	Thorlabs, AC508-500-B
SLM3	Spatial Light Modulator, 1272 x 1024 pixels, 12.5 μm pitch	Hamamatsu, LCOS X13138-07
L10	Lens, focal length = 750 mm	Thorlabs, AC508-750-B
G2	Blazed diffraction grating, 600 lines/mm	Thorlabs, GR50-0610
L11	Lens, focal length = 500 mm	Thorlabs, AC508-500-B
L12	Lens, focal length = 300 mm	Thorlabs, AC508-300-B
Obj	Objective lens, 40X, 0.8 NA, f = 5 mm, water	Nikon, CFI APO NIR
DC	Dichroic mirror, 70 x 50 mm	Semrock, #FF705-Di01
QB	Quad-band filter, 405, 488, 561, 640 nm	Chroma, ZET405/488/561/640
BP	Band-pass filter, 525/50	Chroma ET525/50
SP	Short-pass filter, 2P excitation fluorescence blocker	Semrock #FF01-750sp
LEDs	LED sources, 490 and 430 nm	Thorlabs, M490L4 or M430L5
TL	Tube lens	Thorlabs, TTL200-A
Camera A	sCMOS camera, 6.5 μm pixel, 95 % QE	Photometrics Kinetix
Camera B	sCMOS camera, 6.5 μm pixel, 85 % QE	Hamamatsu ORCA Flash 4.0

Supplementary Table 2: List of optical components of the setup used for 2P voltage imaging with 1030 nm low repetition rate laser (Setup 2)

Component	Description	Manufacturer, Part Reference
Laser E	Femtosecond source, fibre amplifier, fixed output 1030 nm, tuneable repetition rate (0-2 MHz, max power 20 W, 300 fs)	Amplitude, Satsuma HP2
	Pinhole	Thorlabs, SM1D12
L1	Lens, focal length = 300 mm	Thorlabs, AC508-300-B
L2	Lens, focal length = 250 mm	Thorlabs, AC508-250-B
G1	Transmission Grating, 800 lines/mm	GP3508P, Thorlabs
L5	Lens, focal length = 500 mm	Thorlabs, AC508-500-B
SLM2	Spatial Light Modulator, 1920 × 1152 pixels, 9.2 um pixel pitch	HSP1920-600-1300-HSP8, Meadowlark Optics
	Zero order blocker	Hand-made
L6	Lens, focal length = 200 mm	Thorlabs, AC508-200-B
L7	Lens, focal length = 250 mm	Thorlabs, AC508-250-B
L8	Lens, focal length = 19 mm	Thorlabs, AC127-019-B
L9	Lens, focal length = 200 mm	Thorlabs, AC508-200-B
SLM3	Spatial Light Modulator, 1920 × 1152 pixels, 9.2 um pixel pitch	HSP1920-600-1300-HSP8, Meadowlark Optics
L10	Lens, focal length = 500 mm	Thorlabs, AC508-500-B
G2	Blazed diffraction grating, 830 lines/mm	G830R800MG, Optometrics
L11	Lens, focal length = 500 mm	Thorlabs, AC508-500-B
L12	Lens, focal length = 200 mm	Thorlabs, AC508-200-B
Obj	Objective lens, 20X, 1.0 NA, f=10 mm, water immersion	Zeiss, W Plan-Apochromat
DC1	Dichroic mirror	Croma, AT515DC
BP	Band-pass filters, 479/40 and 536/40	Semrock, FF01-479/40-25, FF01-536/40-25
SP	Short-pass filter, 2P excitation fluorescence blocker	Semrock #FF01-750sp
LEDs	LED sources, 490 and 430 nm	pE-4000, CoolLED
TL	Tube lens	TL of Axio Examiner.Z1
Camera A	sCMOS camera, 6.5 µm pixel, 95 % QE	Photometrics Kinetix

Supplementary Table 3: List of optical components of the setup used for in vivo 2P voltage imaging (Setup 3)

Component	Description	Manufacturer, Part Reference
Laser F	Femtosecond source, fibre amplifier, fixed output 1030 nm, tuneable repetition rate (0-2 MHz, max power 50 W, 300 fs)	Amplitude, Satsuma HP3
λ/2	Half-wave plate	Thorlabs, WPH05M-1030
PBS	Polarizing beam splitter	Thorlabs, PBS12-1064
L8	Lens, focal length = -50 mm	CVI-Melles Griot, LPK-25.0-25.9-C
L9	Lens, focal length = 250mm	CVI-Melles Griot, LPX-50.0-129.7-C
SLM3	Spatial Light Modulator, 600 x 800 pixels, 20 μm pitch	Hamamatsu, LCOS 10468-07
L10	Lens, focal length = 500 mm	Thorlabs, AC508-500-B
G2	Blazed diffraction grating, 600 lines/mm	G600R1.0MG, Optometrics
L11	Lens, focal length = 500 mm	Thorlabs, AC508-500-B
L12	Lens, focal length = 300 mm	Thorlabs, AC508-300-B
Obj	Objective lens, 20X, 1.0 NA, f=9 mm, water immersion	XLUMPLFLN20XW, Olympus
DC1	Dichroic mirror	Semrock Di01- R488
BP	Band-pass filter 525/50	Chroma, ET525/50
SP	Short-pass filter, 2P excitation fluorescence blocker	Semrock #FF01-750sp
LEDs	LED source, 470 nm	Thorlabs, M470L2
L13	Lens, focal length = 30 mm	Thorlabs, #LA1805
TL	Tube lens	Thorlabs, TTL200-A
Camera A	sCMOS camera, 6.5 μm pixel, 95 % QE	Photometrics Kinetix

Supplementary Table 4: Experimental configurations

Figure	Panel	Samples	Protein expressed	Temp (°C)	Laser	Rep rate (MHz)	λ (nm)	Method	Spot size (µm)	Average power per cell (mW)	Power density (mW µm^{-2})	Camera	Illumination duration (ms)	Acquisition rate (Hz)	FOV (µm x µm)	z (µm)	N	
**Main text**																		
	b	CHO	JEDI-2P-kv	21 - 23	A	80	940	All	12	100	0.88	A	3000	100	300 x 300	0	17 (GPC), 9 (Gauss), 15 (CGH)	Independent transfections: 3 (GPC), 2 (Gauss), 2 (CGH)
2	d - e	CHO	JEDI-2P-kv	21 - 23	A	80	940	All	12	75 - 175	0.66 - 1.55	A	3 x 200	100	300 x 300	0	12 (GPC), 8 (Gauss), 13 (CGH)	Independent transfections: 2 (GPC), 2 (Gauss), 2 (CGH)
	f	CHO	JEDI-2P-kv	21 - 23	A	80	940	All	12	150 - 175	1.33 - 1.55	A	500	1000	43 x 300	0	11 (GPC), 8 (Gauss), 11 (CGH)	Independent transfections: 2 (GPC), 2 (Gauss), 2 (CGH)
	b - c	Organotypic	JEDI-2P-kv	31 - 35	A	80	940	GPC	12	75 - 175	0.66 - 1.55	A	50 x 10	500 - 1000	43 - 86 x 300	0 - 50	4 - 6	3 slices from 1 slice culture
3	d	Organotypic	JEDI-2P-kv	31 - 35	B	80	920	GPC	12	75 - 175	0.66 - 1.55	B	variable	500 - 1000	43 - 86 x 300	0 - 50	2 - 5	3 slices from 1 slice culture
	e	Organotypic	JEDI-2P-kv	31 - 35	A	80	940	GPC	12	150	1.55	A	30000	1000	43 x 300	0 - 50	> 10	3 slices from 3 independent slice cultures
4		Organotypic	JEDI-2P-kv	31 - 35	B	80	920	GPC	12	125	1,11	B	6 x 40	1000	43 x 300	0 - 50	6	2 slices from 1 slice culture
5		Organotypic	JEDI-2P-kv	21 - 23	E	0.5	1030	Gaussian	17	7.5 - 12.5	0.03 - 0.06	A	30000	500	172 x 300	0 - 50	15	3 slices from 2 independent slice cultures
6		Mouse barrel cortex (L2/3)	JEDI-2P-kv	Body temp	F	0.5	1030	CGH	17	5 - 15	0.02 - 0.07	A	30000	500	172 x 300	<270	43 FOV from 7 mice	Total imaged: 7 mice, 43 FOV, 203 cells
7	c - d	Organotypic	JEDI-2P-kv / ChroME-ST	31 - 35	C	0,25	940	CGH	12	2.5 - 5	0.02 - 0.04	A	5 x 15	1000	43 x 300	0 -50	9 cells	4 slices from 2 independent slice cultures
	e - f	Organotypic	JEDI-2P-kv / ChroME-ST	31 - 35	C	0,25	940	CGH	12	2.5 - 5	0.02 - 0.04	A	5 - 10 x 15	500	86 x 300	0 -50	Up to 10 cells simultaneously	4 slices from 2 independent slice cultures

Supplementary Information																		
Figure	Panel	Samples	Protein expressed	Temp (°C)	Laser	Rep rate (MHz)	λ (nm)	Method	Spot size (µm)	Average power per cell (mW)	Power density (mW μm^{-2})	Camera	Illumination duration (ms)	Acquisition rate (Hz)	FOV (µm x µm)	z (µm)	N	
Supp 2		CHO	JEDI-2P-kv	21 - 23	A	80	940	All	12	100	0.88	A	3000	100	300 x 300	0	41	19 independent transfections
Supp 3		CHO	JEDI-2P-kv	21 - 23	A	80	940	All	12	100	0.88	A	3000	100	300 x 300	0	17 (GPC), 9 (Gauss), 15 (CGH)	Independent transfections: 3 (GPC), 2 (Gauss), 2 (CGH)
Supp 4		CHO	JEDI-2P-kv	21 - 23	A	80	940	All	12	75 - 175	0.66 - 1.55	A	3 x 200	100	300 x 300	0	12 (GPC), 8 (Gauss), 13 (CGH)	Independent transfections: 2 (GPC), 2 (Gauss), 2 (CGH)
Supp 5		CHO	JEDI-2P-kv	21	A	80	940	All	12	150 - 175	1.33 - 1.55	A	500	1000	43 x 300	0	11 (GPC), 8 (Gauss), 11 (CGH)	Independent transfections: 2 (GPC), 2 (Gauss), 2 (CGH)
Supp 6	a - c	Organotypic	JEDI-2P-Kv	31-35	A	80	940	GPC	12	100	0,88	A	3000	100	300 x 300	0 - 50	15	2 slices from 1 slice culture
	d - f	CHO / organotypic	JEDI-2P-Kv	21 - 23 / 31 - 35	A	80	940	GPC	12	100	0.88	A	3000	100	300 x 300	0 - 50	17 (CHO), 15 (organotypic)	3 independent transfections (CHO), 2 slices from 1 slice culture
	h	Organotypic	JEDI-2P-Kv	31 - 35	A	80	940	GPC	12	150	1.33	A	10	1000	43 x 300	0 - 50	5	2 slices from 1 slice culture
Supp 7	a - c	Organotypic	JEDI-2P-Kv	31 - 35	B	80	940	GPC	12	75 - 175	0.66 - 1.55	B	variable	500 - 1000	43 - 86 x 300	0 - 50	2 - 5	3 slices from 1 slice culture
	d	Organotypic	JEDI-2P-Kv	31 - 35	B	80	920	GPC	12	125	1,11	B	6 x 40	1000	43 x 300	0 - 50	6	2 slices from 1 slice culture
Supp 8	b - g	Organotypic	JEDI-2P-Kv	31 - 35	A	80	940	CGH	12	75 - 200	0.66 - 1.77	A	6 x 30000	1000	43 x 50	0 - 50	5 - 6	5 slices from 4 independent slice cultures
	h	Organotypic	JEDI-2P-kv	31 - 35	A	80	940	CGH	12	125	1.11	A	10 x 30000	500	86 x 300	0 - 50	3	2 slices from 1 slice culture
	i	Organotypic	JEDI-2P-kv	31 - 35	A	80	940	CGH	12	75 - 200	0.66 - 1.77	A	6 x 30000	1000	43 x 50	0 - 50	5 - 6	5 slices from 4 independent slice cultures
Supp 10	b	CHO	JEDI-2P-kv	21 - 23	A	80	940	GPC	12	100	0.88	A	15 x 400	100	300 x 300	0 - 50	8	1 transfection
	c	Organotypic	JEDI-2P-kv	31 - 35	A	80	940 / 1030	CGH no TF	12	150 / 137	1.33 / 1.21	A	50 x 10	1000	43 x 300	0 - 50	4	2 slices from 1 slice culture

Figure	Panel	Samples	Protein expressed	Temp (°C)	Laser	Rep rate (MHz)	λ (nm)	Method	Spot size (µm)	Average power per cell (mW)	Power density (mW µm^{-2})	Camera	Illumination duration (ms)	Acquisition rate (Hz)	FOV (µm x µm)	z (µm)	N	
Supp 10	d - e	CHO	JEDI-2P-kv	21 - 23	D	40	1030	CGH	12	20 - 98	0.18 - 0.87	A	6000	5	300 x 300	0 - 50	6	1 transfection
	f	Organotypic	JEDI-2P-kv	31 - 35	F	0.25 - 2	1030	CGH	17	6 - 21	0.03 - 0.09	A	30000	500	172 x 300	0 - 50	27 - 50	3 - 5 slices from 1 slice culture
Supp 12	b - g	Organotypic	JEDI-2P-kv	21 -23	E	1	1030	CGH	12	5 - 30	0.04 - 0.27	A	6 x 30000	-	-	0 - 50	5 - 6	6 slices from 2 independent slice cultures
	h	Organotypic	JEDI-2P-Kv	31 - 35	D	40	1030	CGH	12	75	0,66	A	7 x 30000	500	86 x 300	0 - 50	3	2 slices from 1 slice culture
Supp 13		Organotypic	JEDI-2P-kv	21 - 23	E	0.5	1030	Gaussian	17	7.5 - 12.5	0.03 - 0.06	A	30000	500	172 x 300	0 - 50	15	3 slices from 2 independent slice cultures
Supp 14		Mouse barrel cortex (L2/3)	JEDI-2P-kv	Body temp	F	0.5	1030	CGH	17	5 - 15	0.02 - 0.07	A	30000	500	172 x 300	46 - 270	43 FOV from 7 mice	Total: 7 mice, 43 FOV, 203 cells
Supp 16		Organotypic	JEDI-2P-kv / ChroME-ST	31 - 35	C	0.25	940	CGH	12	2.5 - 9	0.02 - 0.08	A	5 - 10 x 15	500	86 x 300	0 - 50	Up to 10 cells simultaneously	4 slices from 2 independent slice cultures

Supplementary Table 5: Experimental Protocols

Protocol No	Electrical Stimulation				Laser Illumination			Schematic representation
		**No. pulses**	**Pulse duration (ms)**	**Frequency/ Interpulse time (Hz/ ms)**	**No. Ill. Epochs**	**Ill. epoch duration (ms)**	**Inter-epoch duration (ms)**	
1	100 mV Voltage steps	3	100	0.4 /2700	1	3000	-	**Protocol 1** 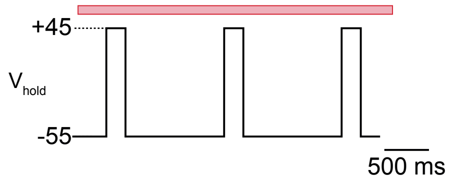
2	100 mV Voltage steps	3	100	0.4 /2700	3	200	2500	**Protocol 2** 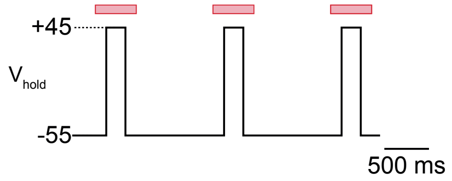
3	100 mV Voltage steps	10	3	20/47	1	500	-	**Protocol 3** 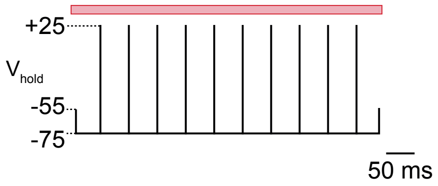

4	200-400 pA current injection	5	10	5/190	1	3000	-	**Protocol 4** 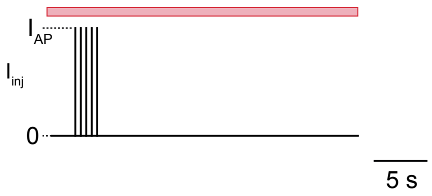
5	200-500 pA current injection	random	5	random	1	3000	-	**Protocol 5** 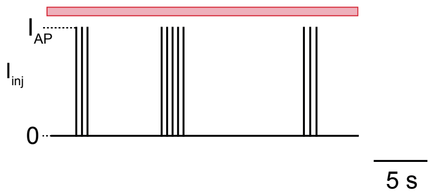

Supplementary Table 6: Comparative table of 2P optical approaches

Illumination method	Indicator	Max depth (µm)	Laser power	Spot size Diameter* (µm)	Laser Peak Intensity (GW mm^{-2})	Longest imaging recording	Reference
2P microscope in linescan mode	ANNINE-6plus	50	60 mW	1	7,1	4 min	[1]
2P raster scanning on a rectangular field-of-view around the cell	ASAP1, CAESR, ArcLight	-	4 mW	1	0,5	25 seconds	[2]
2P scanning	di-3-ANEPPDHQ	150	10–20 mW	1	1,6	1 second	[3]
FACED (2P)	ASAP3	345	10-85 mW	1 (80 beamlets)	4,2	6 seconds	[4]
ULoVE (2P)	ASAP3	440	20 mW/cell on the target	1 (3 spots considered)	2,3	150 seconds	[5]
RAMP (AODs) (2P)	ASAP2s	130	5-30 mW	1	4,8		[6]
Spatiotemporal Multiplexing	SpikeyGi/2	300	30 mW/beamlet (240 mW for 8 beamlets)	1 (8 beamlets)	12,2	1 h	[7]
ULoVE (2P)	JEDI-2P	430	max 30 mW/cell on the target	1 (2 spots considered)	4,8	40 min	[8]
2P-scanning	ASAP4	185	18-31 mW	1	1,7	100 s, 10's of minutes	[9]
Parallel illumination with TF	JEDI-2P	250	7,5 mW/cell	17	0,22	30 s	This work

*We consider a near-diffraction-limited spot of ~1 µm diameter for all methods, since this is not specified differently in none of the works cited.

Supplementary Method 1 – Extracting fluorescence timeseries from scanless 2P voltage imaging data

Accompanies discussion and data for Figure 2 of the main text and Supplementary Figures 2 - 6.

A data analysis pipeline was developed based on existing routines[10–12]. The pipeline was tested on simulated data and refined on data collected from CHO cells with electrophysiological ground truth data (whole cell patch clamp). The data analysis pipeline was written in Python with SciPy, NumPy and Scikit-Image dependencies.

1. For single target datasets proceed to step 2. For multi-cell datasets, check whether any overlap between target ROIs (usually 175 pixels x 175 pixels per target),

    if overlap:
    a. Manually segment the target index and proceed directly to step 2f
    else:
    a. crop each cell using the camera co-ordinates of the targeted spot (175 pixels x 175 pixels)

2. For cropped data:
    a. Calculate neighbourhood spatiotemporal correlation (8x8 pixel neighbourhood)
    b. Create an initial binary image using Otsu thresholding on the 2D image computed in step 2 (using inbuilt *threshold_otsu* function from Python library scikit-image)
    c. Segment the binary image using a random walker algorithm. Remove holes and unconnected pixels in the segmented image. (using inbuilt *random_walker* function from Python library scikit-image, beta parameter set to 130 and 'cg' mode).
    d. Clean segmentation (using inbuilt *binary_fill_holes* function from Python library SciPy, and inbuilt functions *erosion*, *dilation* and *disk* from Python library scikit-image, with disk radius 1.2 times the average cell diameter.
    e. Retain the segment containing the largest number of connected pixels (using inbuilt function *label* from the Python library scikit-image).
    f. Calculate an initial fluorescence trace as the average value of each acquired frame multiplied by the binary segmentation mask. This trace is referred to as the "unweighted trace" throughout this work.
    g. Calculate the background fluorescence trace as the average value of each acquired frame multiplied by the pixels outside of the segmentation mask computed in step 2c.
    h. If necessary:
    Detrend initial fluorescence trace and original stack with a high-pass filter to remove any slow variations (such as slow fluctuations in membrane potential or photobleaching). Inbuilt functions *butter* and *filtfilt* from Python library SciPy), were used; "cut on" frequency set as a function of imaging rate (e.g., for 100 Hz imaging, 0.3 Hz "cut on" used).
    i. Generate a spatial filter by ridge regression of the (detrended) trace against the (detrended) stack as per reference[10].
    j. Calculate the final fluorescence trace as the weighted spatial average of the segmented pixels. A summary of this procedure is presented in Supplementary Figure 2.

As demonstrated in Supplementary Figures 2-6, compared with results generated by calculating the unweighted mean of pixels within segmented cells, the regression-based pixel weighting algorithm improved -%$\Delta F/F_0$ with a minor increase in photobleaching, without having a significant impact on SNR. We hypothesise the increase in photobleaching is the result of voltage responsive fluorophores are more likely to be tethered to the membrane and hence less mobile. Based on the results presented in Supplementary Figures 2-6, this pipeline was used to extract fluorescence timeseries from scanless 2P voltage imaging data with camera detection for subsequent analysis.

Furthermore, as described in reference[10], template matching was used to identify putative action potentials (APs) in relevant traces.

Supplementary Method 2 – Calibration procedure for multi-cell experiments

In order to target excitation spots to specific locations in the field of view, it is necessary to estimate the mapping between “camera” and “SLM” co-ordinates. For all modalities (TF-CGH/ TF-GPC/ TF-Gauss), this was achieved prior to all experiments as follows:

1. A thin spin-coated rhodamine layer was placed in the focal plane of the microscope.
2. A single spot was generated at the optical axis (the origin of the SLM coordinate system). 10 images were recorded and averaged.
3. A single spot was generated at the edge of the desired FOV. 10 images were recorded and averaged.
4. A grid of spots spanning the desired FOV was generated (usually 5x5 spots). 10 images were recorded and averaged.
5. The locations of the spots in the images acquired in steps 2-4 (in camera coordinates) were estimated using built-in circle detection functions of scikit-image[13].
6. The affine transformation between “camera” and “SLM” co-ordinates was calculated based on the estimated positions in step 5. This transformation was used to generate SLM co-ordinates to target spots to cells.

Prior to experiments, the calibration procedure was refined by adding and localizing spots in random locations throughout the field of view until the error in spot position was less than 1 camera pixel.

The data acquired in step 4 was also used to generate a diffraction efficiency map which was used to tune the power delivered to cells in different portions of the field of view.

This calibration procedure was repeated daily to ensure accurate targeting in all experiments and to measure the stability of the system alignment.

Supplementary Figure 1

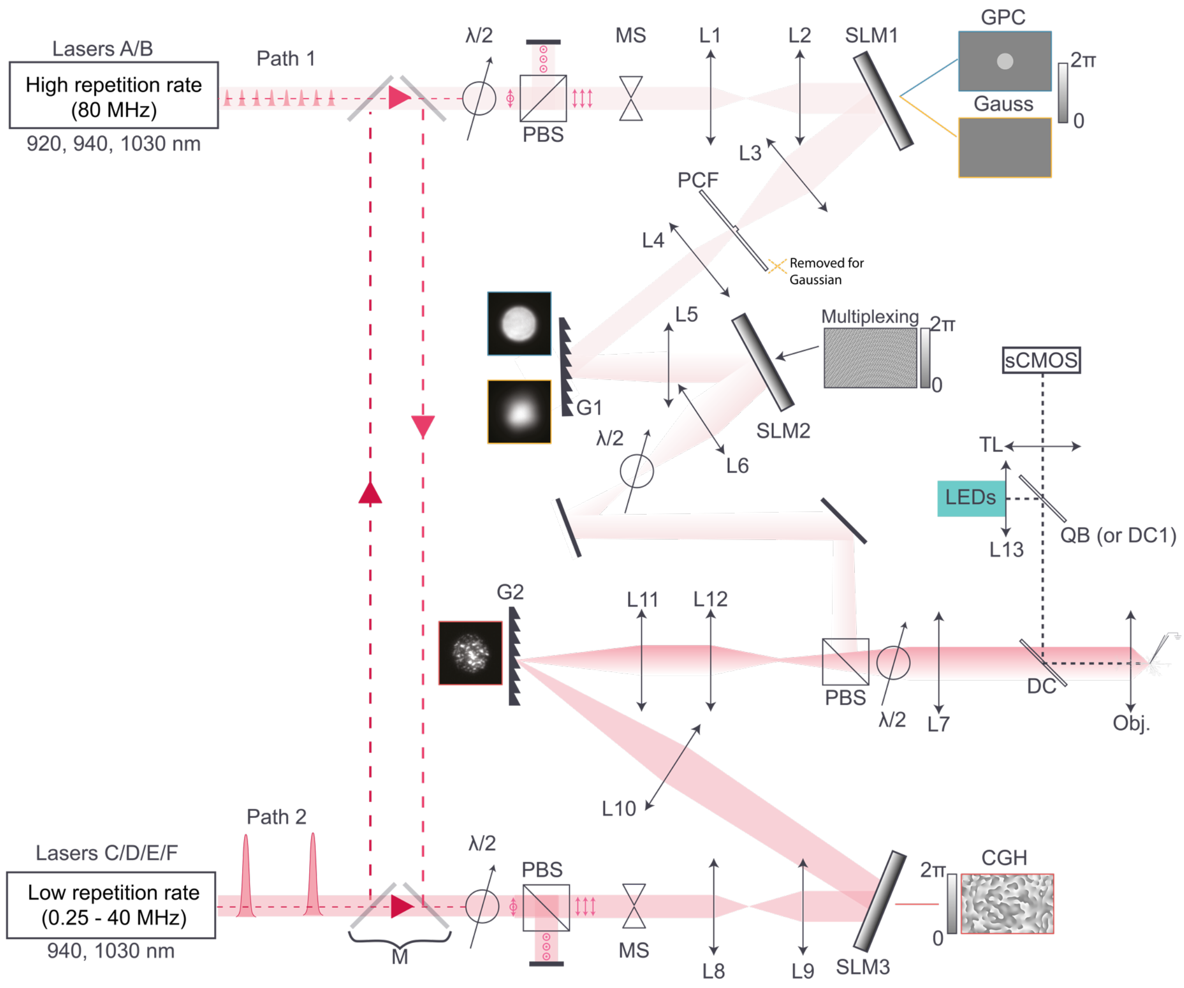

Supplementary Figure 1 - Optical setup for scanless 2P voltage imaging

Accompanies Figure 1 of the main text and Supplementary Tables 1-3.

Schematic diagram of the optical setup designed to generate 12-17 μm (FWHM), temporally focused (TF), Generalised Phase Contrast (GPC), Gaussian (Gauss) and Computer-Generated Holography (CGH) spots. The setup was equipped with different high and low repetition rate lasers (refer to Supplementary Table 1 for details). Gaussian and GPC spots were generated using Path 1 (upper, as indicated) and spatially multiplexed using a second spatial light modulator, SLM2. CGH spots were generated using path 2 (lower, as indicated), where an expanded beam was sent to a spatial light modulator addressed with a computer-generated phase profile. Paths 1 and 2 were combined prior to the objective (Obj.) with a polarising beam splitter (PBS). Acronyms: λ/2 (half-wave plate), MS (Mechanical Shutter,), L (Lens), SLM (Spatial Light Modulator), PCF (Phase Contrast Filter), G (Grating), DC (Dichroic), QB (Quad-band filter), TL (Tube Lens). For data presented in Figure 5, Supplementary Figures 10c-e, and 12 refer to Supplementary Table 2 for the optical components used. For experiments of Figures 6 and Supplementary Fig. 10f, 13 and 14 refer to Supplementary Table 3.

Supplementary Figure 2

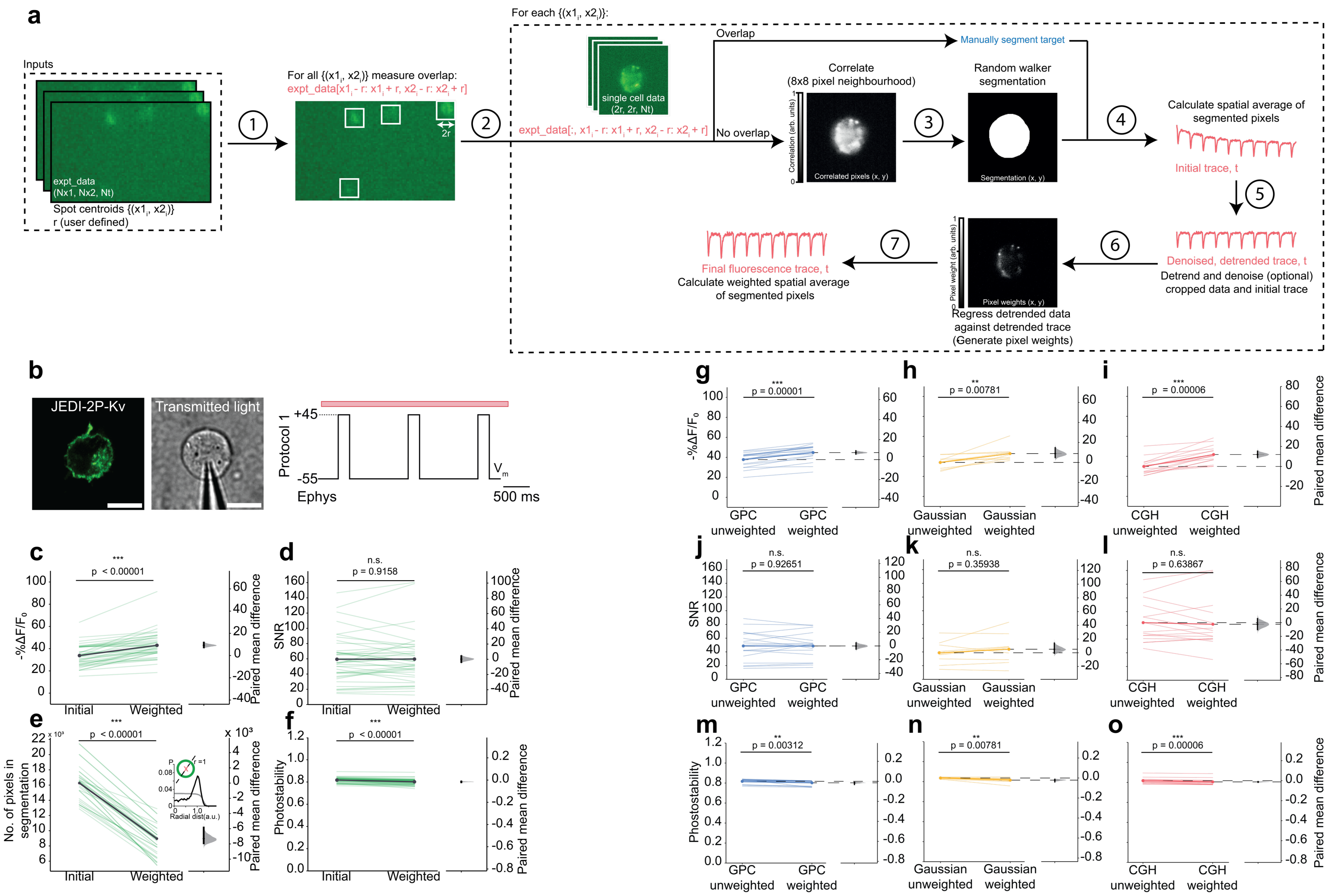

Supplementary Figure 2 - Summary and evaluation of the data analysis pipeline used for scanless 2P voltage imaging

Accompanies Figures 2 - 5 of the main text.

(a) Overview of the data analysis pipeline used to generate fluorescence traces from scanless 2P imaging data. Refer to Supplementary Method 1 for more details. **(b)** Schematic representation of experimental protocol used to evaluate the data analysis pipeline (Protocol 1, Supplementary Table 5). Cross-section through a confocal stack of a JEDI-2P-Kv expressing CHO cell (left) and transmitted light image of a patched cell (middle). Scale bars represent 10 μm. The electrophysiology protocol used during data acquisition is plotted in black (right). The red bar above the electrophysiology trace indicates the illumination epoch (3 s). **(c-f)** Quantitative comparison demonstrating the utility of applying a regression-based approach to extract fluorescent time series from scanless 2P voltage imaging data (weighted) in contrast to simple segmentation (initial). Each line in each plot represents a single measurement from a single cell, the mean of all measurements is plotted in black (n = 41 cells, 19 independent transfections). **(c-f)** Paired comparisons of **(c)** -%$\Delta F/F_0$, **(d)** SNR, **(e)** number of pixels per mask between traces generated using the initial or weighted segmentations and associated Gardner-Altman plots (see Methods and reference[5]). **(e)** (inset) The average radial probability distribution functions of pixels used in the weighted and unweighted (initial) masks**.** In the case of the weighted mask, the distribution function is strongly peaked at the cell membrane indicating that the analysis pipeline successfully identified pixels which recorded membrane localized (and hence voltage-sensitive) fluorescence. **(f)** Paired comparison and Gardner-Altman plot of the photostability of traces generated using the initial or weighted segmentations. **(g-o)** Paired comparison (and associated Gardner-Altman plots) of **(g-i)** -%$\Delta F/F_0$ **(j-l)** SNR and **(m-o)** photostability between simple segmentation (unweighted data) and regressed (weighted) data for each of the different modalities; TF-GPC (blue), TF-Gaussian (yellow) and TF-CGH (red). Each line represents a single measurement from an individual cell. The average values are plotted in a darker shade of the same colour, n = 10-20 cells for all modalities. * Denotes p<0.05, ** denotes p<0.01 and *** denotes p<0.0001 (t-test, see Methods).

Supplementary Figure 3

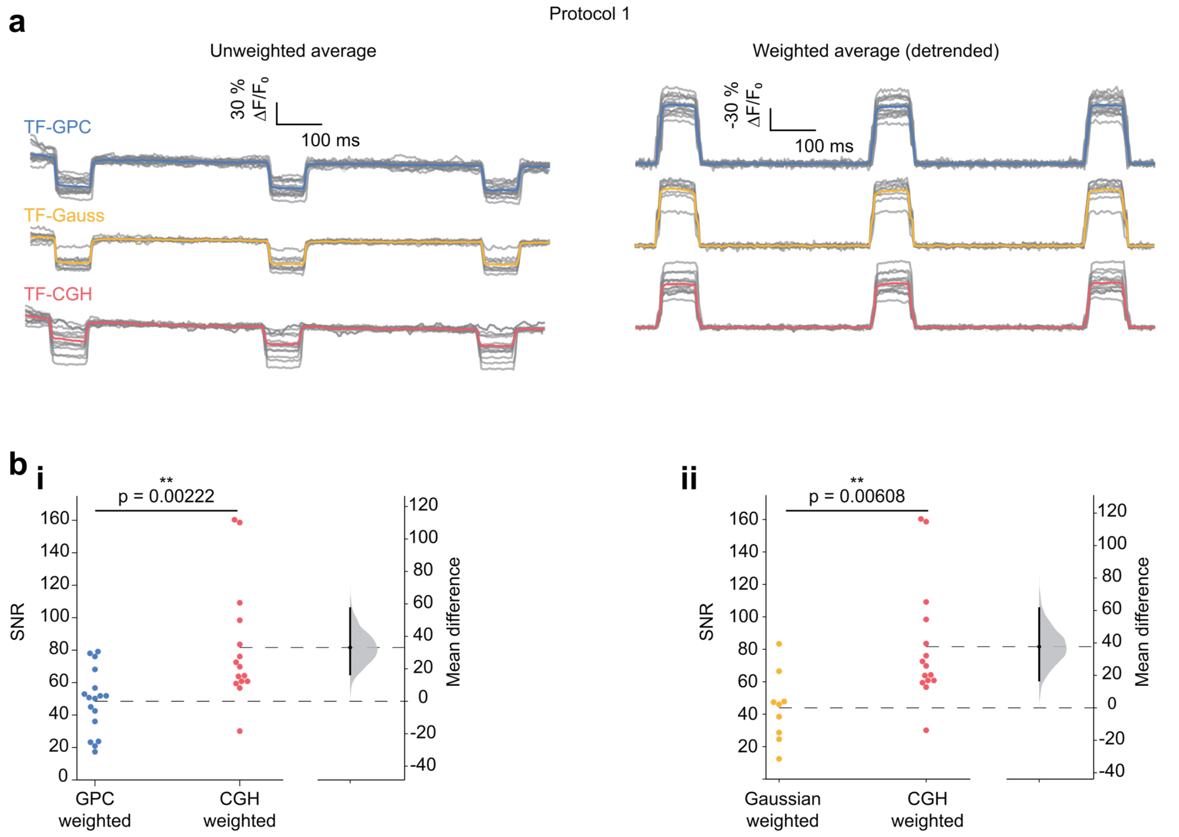

Supplementary Figure 3 - Comparison of different parallel illumination approaches for scanless 2P voltage imaging (protocol 1)

This figure accompanies Figure 2 of the main text and Supplementary Figure 2.

(a) Unweighted %$\Delta F/F_0$ (left) and processed (weighted/detrended) -%$\Delta F/F_0$ (right) fluorescence traces from single cells (individual trials, grey) and their average (TF-GPC (blue), TF-Gaussian (yellow) and TF-CGH (red)) for protocol 1 (Supplementary Table 5). Refer to Supplementary Method 1 and Supplementary Figure 2 for a description of the difference between weighted and unweighted traces. **(b)** Comparison and Gardner-Altman plot of SNR between the three different modalities for protocol 1; (i) TF-GPC (blue) vs TF-CGH (red) (ii) TF-Gaussian (yellow) vs TF-CGH (red) (n = 9 - 17 cells, 2 - 3 independent transfections per modality). The results are consistent with those found for Protocol 2, discussed in detail in results section of the main text. * denotes p<0.05, ** denotes p<0.01 and *** denotes p<0.0001 (t-test, see Methods).

Supplementary Figure 4

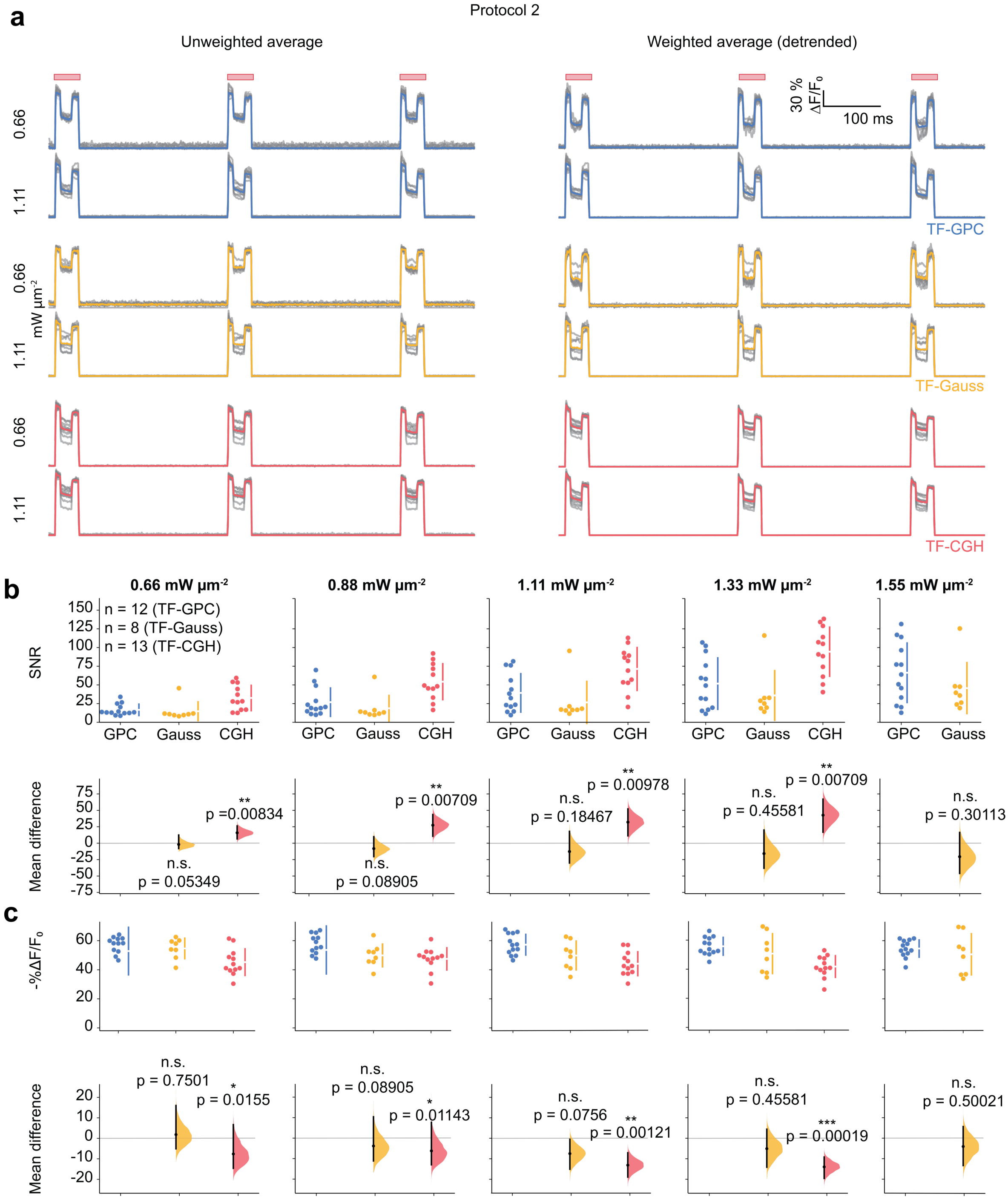

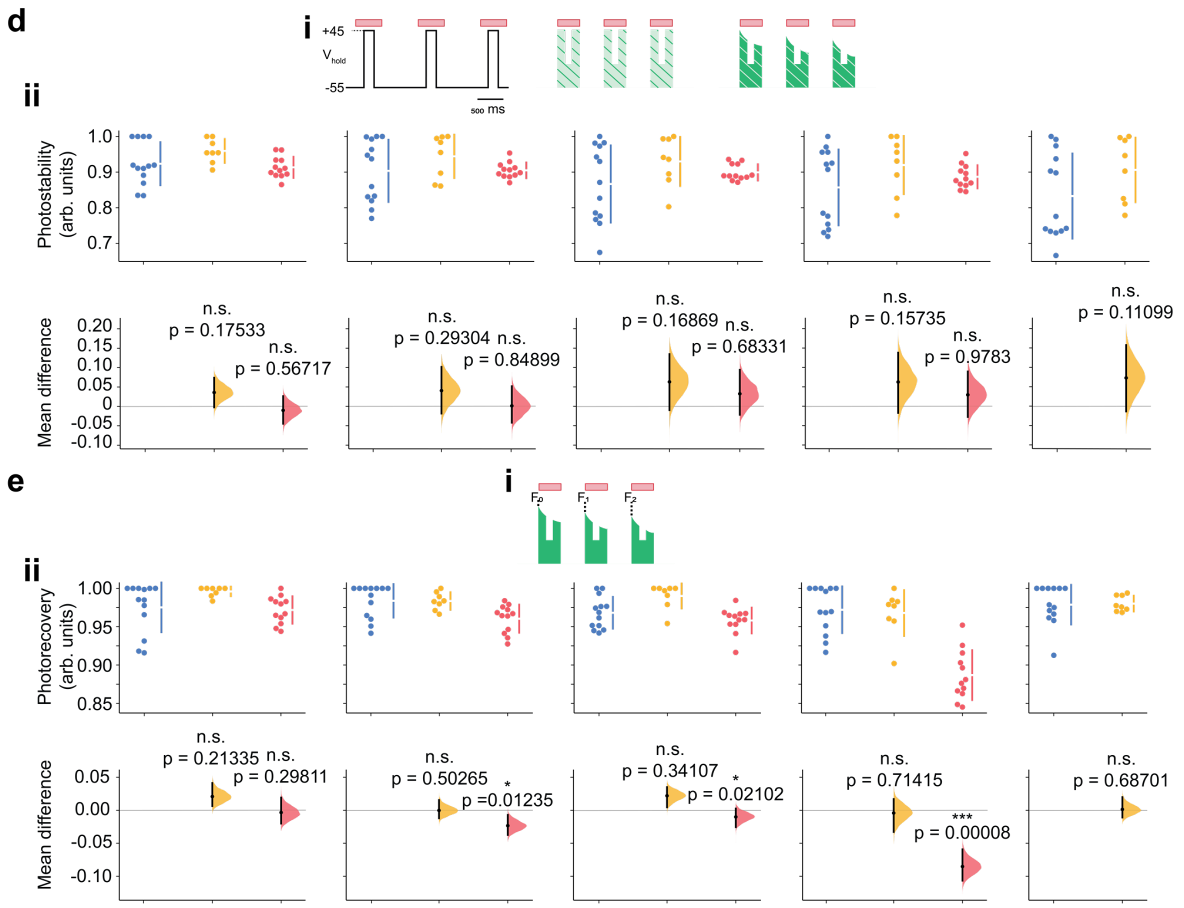

Supplementary Figure 4 - Comparison of different parallel illumination approaches for scanless 2P voltage imaging (protocol 2)

This figure accompanies Figure 2 of the main text and Supplementary Figure 2.

(a) Unweighted (left) and weighted (right) %$\Delta F/F_0$ fluorescence traces from single cells (individual trials, grey). The average trace for all cells acquired at a given power using a given modality is plotted in a solid colour (TF-GPC (blue), TF-Gaussian (yellow) and TF-CGH (red)) for protocol 2 (laser A). Refer to Supplementary Method 1 and Supplementary Figure 2 for a description of the differences between weighted and unweighted traces. Traces from two different power densities (0.66, upper and 1.11 mW µm^{-2}, lower, as indicated, corresponding to 75 mW and 125 mW, respectively) are plotted for each modality, as labelled. **(b-d)** Comparison of parallel illumination approaches for scanless 2P voltage imaging obtained using protocol 2 (Supplementary Table 5). **(b)** -%$\Delta F/F_0$, **(c)** SNR, **(d)** photostability and **(e)** photorecovery for each of the different modalities (TF-GPC (blue), TF-Gaussian (yellow) and TF-CGH (red)), at power densities ranging between 0.66 and 1.55 mW µm^{-2}, as labelled (corresponding to 75 – 175 mW per cell). Individual points represent measurements from individual cells. (n = 8 - 13 cells, 2 independent transfections per modality), * denotes p<0.05, ** denotes p<0.01 and *** denotes p<0.0001 (t-test, see Methods). Photostability is defined as the fraction of measured fluorescence to the ideal case of no photobleaching (the ratio of the dark green area to the light green area as depicted in (d)(i)). Photorecovery is defined as the average fraction of measured fluorescence at the start of a given illumination epoch to that at the start of the prior illumination epoch (the average of F_1/F_0 and F_2/F_1 as indicated in (e)(i)).

Supplementary Figure 5

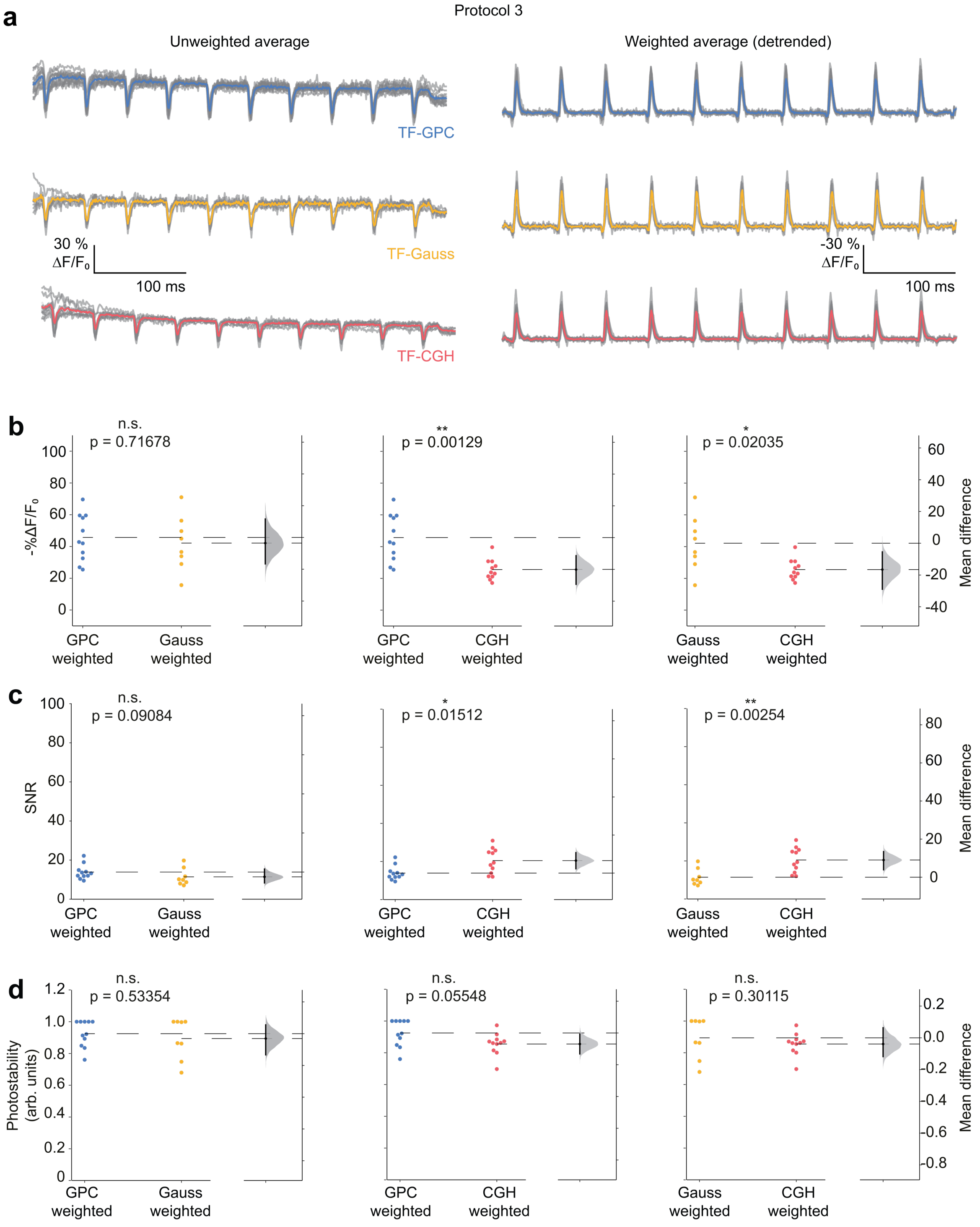

Supplementary Figure 5 - Comparison of different parallel illumination approaches for scanless 2P voltage imaging (protocol 3)

This figure accompanies Figure 2 of the main text, and Supplementary Figure 2.

Comparison of parallel illumination approaches for scanless 2P voltage imaging obtained using protocol 3, as described in the main text (Refer to Supplementary Table 5). **(a)** Unweighted %$\Delta F/F_0$ (left) and processed (weighted/de-trended) -%$\Delta F/F_0$ (right) fluorescence traces from single cells (individual trials, grey) and their average (coloured, TF-GPC (blue), TF-Gaussian (yellow) and TF-CGH (red)) for protocol

3 (laser A). Refer to Supplementary Method 1 and Supplementary Figure 2 for a description of the differences between weighted and unweighted traces. Comparison and Gardner-Altman plots of **(b)** - $\%\Delta F/F_0$, **(c)** SNR and **(d)** photostability between each of the different modalities; TF-GPC (blue), TF-Gaussian (yellow) and TF-CGH (red) (power density: 1.33 mW μm^{-2}, 150 mW per cell, 1 kHz acquisition rate, n = 8 - 11 cells, 2 independent transfections per modality). The results are consistent with those found for Protocol 2, as discussed in the results section of the main text. However, these results were obtained in a low photon flux regime, as the data was acquired at 1 kHz. Since, for all modalities, the SNR is > 11 for an AP-like event we conclude that all modalities can be used to detect single APs in single trials using scanless 2P voltage imaging.

Supplementary Figure 6

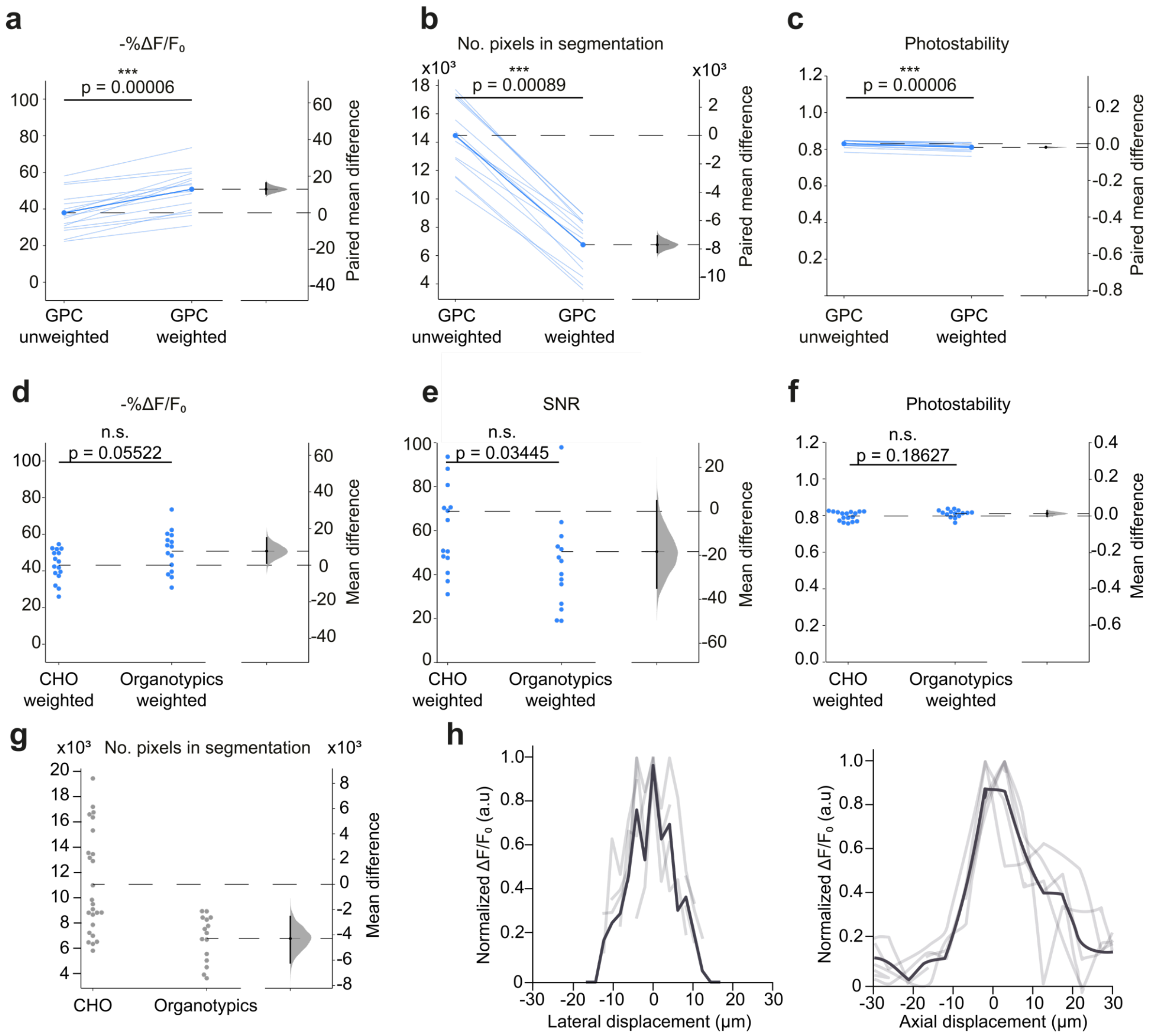

Supplementary Figure 6 - Comparison of scanless 2P voltage imaging between isolated CHO cells and neurons in the densely labelled dentate gyrus region of hippocampal organotypic slices

This figure accompanies Figures 2 and 3 of the main text.

(a-c) Paired comparison and Gardner-Altman plots of (a) -%$\Delta F/F_0$, (b) number of pixels in segmentation and (c) photostability of all cells in response to protocol 1 (Supplementary Table 5) (laser A), in hippocampal organotypic slices, using 2P, TF-GPC (power density: 0.88 mW μm^{-2}, 100 mW per cell, 100 Hz acquisition rate). Each line represents data from an individual trial in individual cells (n = 15 cells). **(d-g)** Comparison of data obtained using protocol 1 between densely labelled hippocampal organotypic slices and CHO cells (and corresponding Gardner-Altman plots). (d) -%$\Delta F/F_0$, (e) SNR, (f) photostability and (g) number of pixels in segmentation. The results demonstrate that the performance of the method does not deteriorate in densely labelled slices due to the axial sectioning conferred by temporal focusing, and top-hat nature of GPC light shaping. Each point represents data from an individual cell. (n = 15 - 17 cells). **(h)** Physiological lateral and axial resolution profiles, quantified as the relative $\Delta F/F_0$ of an electrically evoked spike as a function of the distance between the excitation spot and the soma (14 µm lateral and 13 µm axial FWHM respectively) (n = 5 cells, 2 slices from 1 slice culture). Results from individual trials are plotted in grey and average results are plotted in black.

Supplementary Figure 7

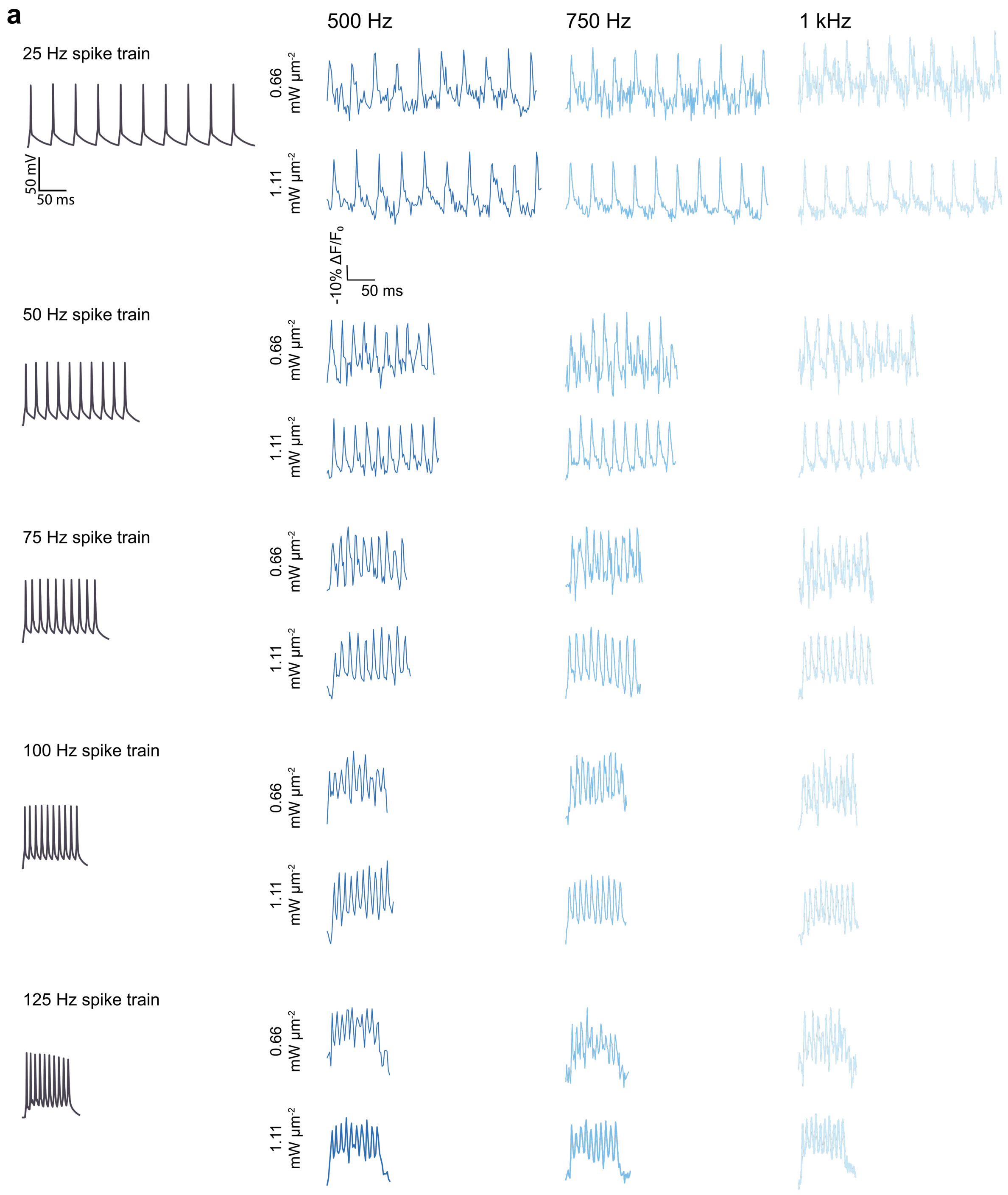

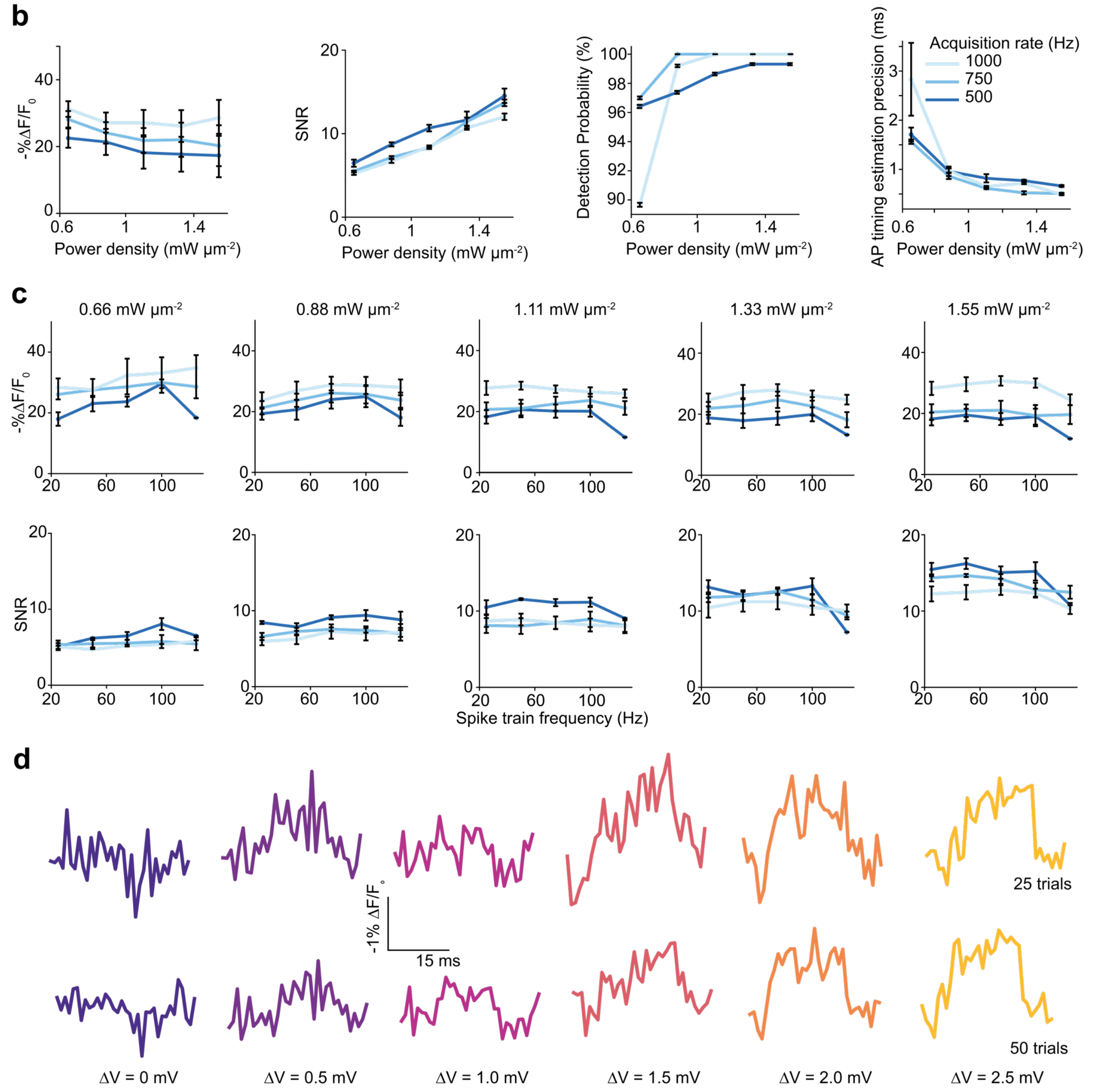

Supplementary Figure 7 - Characterisation of scanless 2P voltage imaging for imaging trains of action potentials and subthreshold activity

This figure accompanies Figures 3 and 4 of the main text.

(a) Representative fluorescence traces recorded from an individual (representative) cell to different rates of electrically evoked spike trains recorded at different acquisition rates plotted in different shades of blue (see legend) (power density: 0.66 mW µm^{-2} in all cases, 75 mW per cell, laser A). **(b)** -%ΔF/F_0, SNR, AP detection probability and precision of AP timing estimation (defined as the jitter in timing estimation for all identified APs relative to the corresponding electrophysiological recordings) plotted as a function of power density for different acquisition rates (500 Hz, 750 Hz, and 1 kHz, see legend). A lower value indicates superior timing estimation. Data plotted for all train rates (n = 2 - 5 cells, from 3 different slices from 1 slice culture). **(c)** -%ΔF/F_0 and SNR plotted as a function of AP train rate for different acquisition rates (500 Hz, 750 Hz, 1 kHz, see legend) and power densities (as labelled). **(d)** Representative fluorescence traces recorded from an individual cell to different steps of subthreshold depolarizations (0 – 2.5 mV, as indicated), after averaging 25 trials (upper) or 50 trials (lower). Data acquired at 1.1 mW µm^{-2} (125 mW per cell), and recorded at 1 kHz.

Supplementary Figure 8

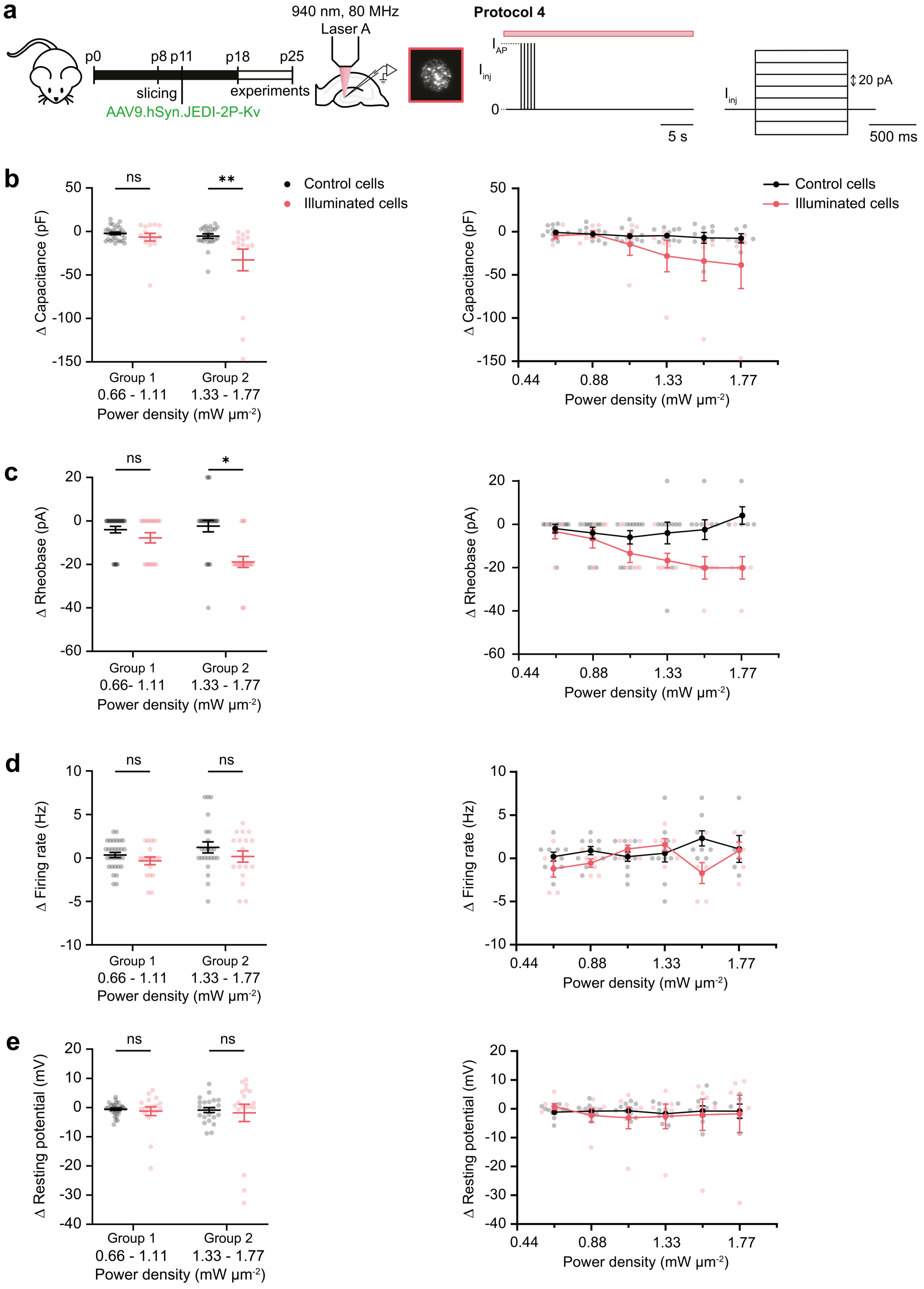

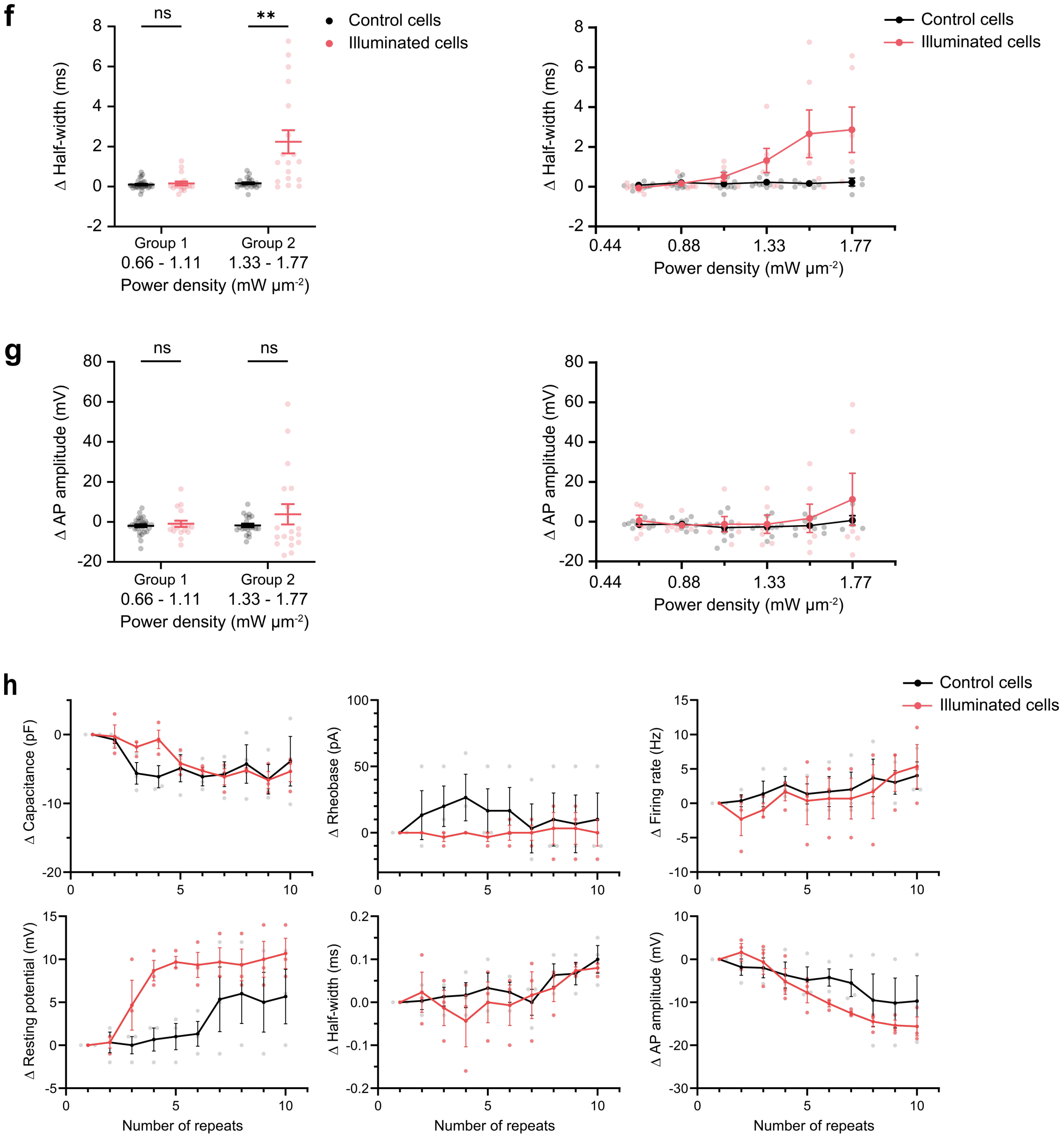
f
ns
**
Control cells
Illuminated cells
Δ Half-width (ms)
Group 1
0.66 - 1.11
Group 2
1.33 - 1.77
Power density (mW μm-2)
g
ns
ns
Δ AP amplitude (mV)
Group 1
0.66 - 1.11
Group 2
1.33 - 1.77
Power density (mW μm-2)
h
Control cells
Illuminated cells
Δ Capacitance (pF)
Δ Rheobase (pA)
Δ Firing rate (Hz)
Δ Resting potential (mV)
Δ Half-width (ms)
Δ AP amplitude (mV)
Number of repeats

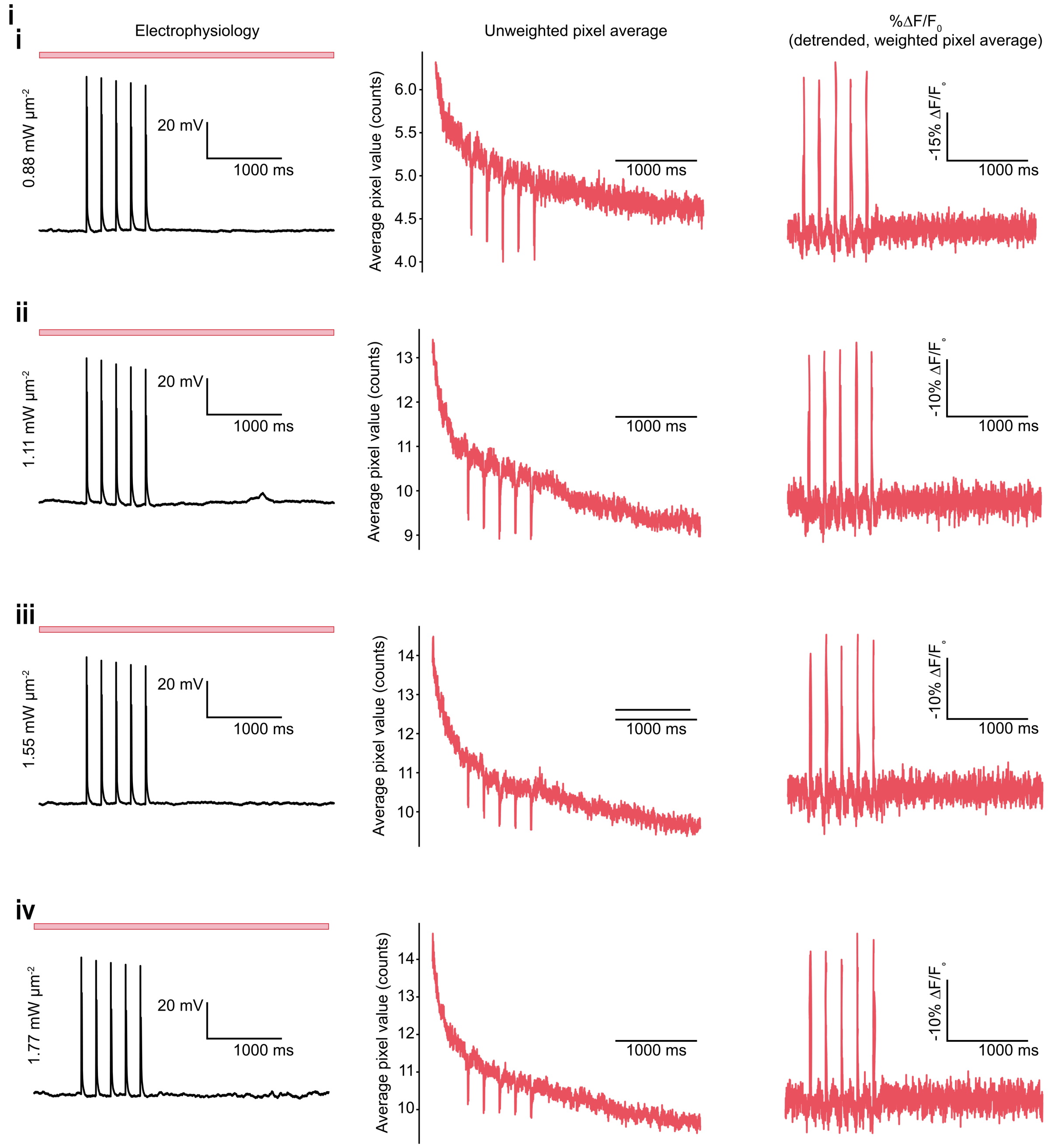

Supplementary Figure 8 - Quantification of perturbations induced by scanless 2P voltage imaging upon illumination with laser pulses at high repetition rate

This figure accompanies Figure 3 of the main text.

(a) Schematics of the experimental protocol (for more details, refer to Methods). **(b - g)** Relative changes in (b) capacitance, (c) rheobase, (d) firing rate, (e) resting potential, (f) AP half-width and (g) AP amplitude as measured using whole-cell patch clamp electrophysiology are plotted as a function of power (protocol 4, see Methods and Supplementary Table 5, red points). The black points are control cells which were patched but not illuminated. Each point represents data from an individual trial. (Left) Data from different cells and power densities have been pooled into two groups (group 1: 0.66 – 1.11 mW µm^{-2} and group 2: 1.33 – 1.77 mW µm^{-2}, corresponding to powers of 75 - 125 mW and 150 - 200 mW, laser A or B) (n = 5 - 6 cells, from 5 different slices from 4 slices cultures). The population average and standard error are indicated in black or red in each case. * denotes p<0.05, ** denotes p<0.01 and

*** denotes $p<0.0001$ (Mann-Whitney *U*-test). (Right) Data from individual cells are plotted as a function of average incident power density (0.66 - 1.77 mW μm^{-2}, powers per cell: 75 – 200 mW) used for scanless 2P imaging (protocol 4, see Methods and Supplementary Table 5, red points). Each point represents data from a single trial. **(h)** Relative changes in capacitance, rheobase, firing rate, resting potential, AP half-width and AP amplitude as measured using whole-cell patch clamp electrophysiology are plotted as a function of number of repeats (power density: 1.11 mW μm^{-2}, power per cell: 125 mW). Each repeat corresponds to 30 s illumination, after which the membrane properties were measured (n = 3 neurons, from 2 different slices from 1 slice culture). **(i)** Representative electrophysiological (left) and fluorescence traces (unprocessed, middle and processed, right) of 5 electrically induced APs recorded at power densities of 0.88 (i), 1.11 (ii), 1.55 (iii) and 1.77 mW μm^{-2} (iv) (corresponding to 100, 125, 175 and 200 mW per cell, respectively). All data were acquired using laser A tuned to 940 nm and camera A (Refer to Supplementary Figure 1 and Supplementary Tables 1 and 2).

Supplementary Figure 9

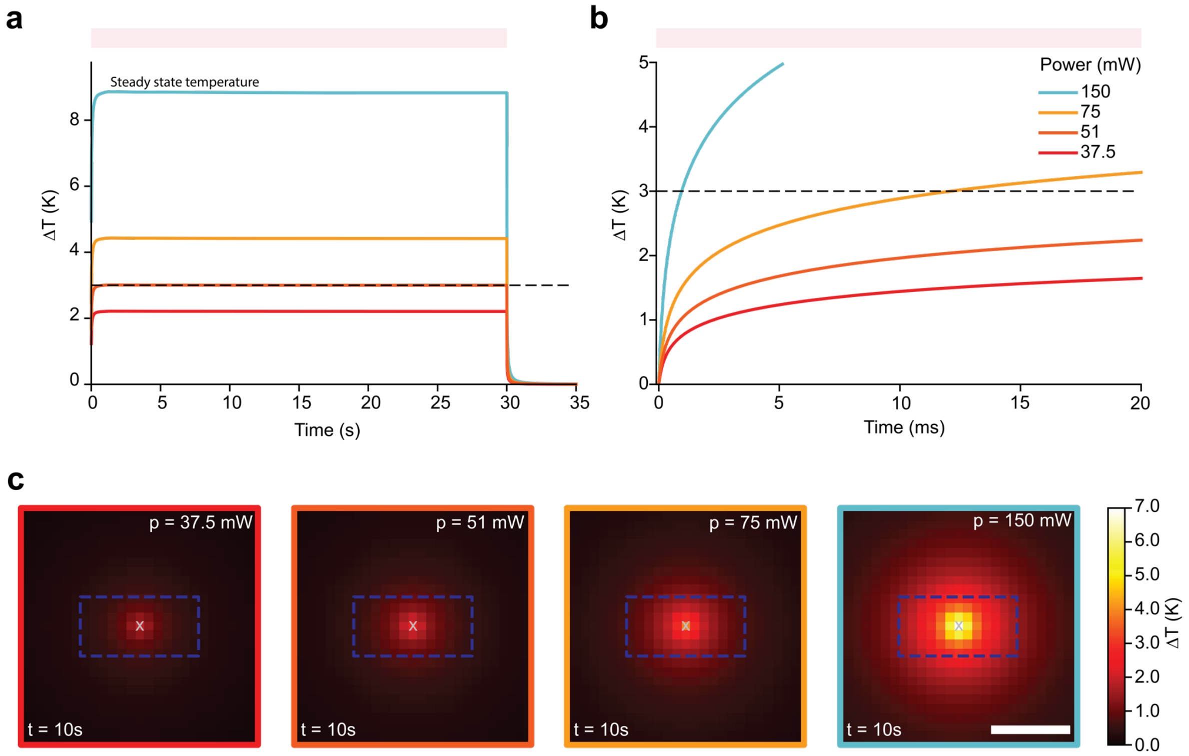

Supplementary Figure 9 – Simulations of temperature rises in tissue upon scanless voltage imaging of single cells with high repetition rate lasers

This figure accompanies Figure 3 of the main text.

(a-b) Temperature rise as a function of time for different illumination powers used for scanless 2P voltage imaging of single neurons in superficial layers. Results from different illumination powers are plotted in different colours (refer to the legend in b). The black dashed line indicates a 3 K temperature rise. The simulation parameters were chosen to match the experimental parameters as closely as possible: a single 17 µm holographic spot centred in the field of view, 940 nm excitation, average powers as specified. **(c)** Spatial profiles of the temperature rises induced by scanless 2P voltage imaging at different average illumination powers (indicated top right) after 10 s. The dashed blue boxes indicate the imaging field of view corresponding to a 500 Hz acquisition rate. In each case, the illuminated neuron (simulated) was located in the centre of the field of view. Scale bar represents 150 µm.

Supplementary Figure 10

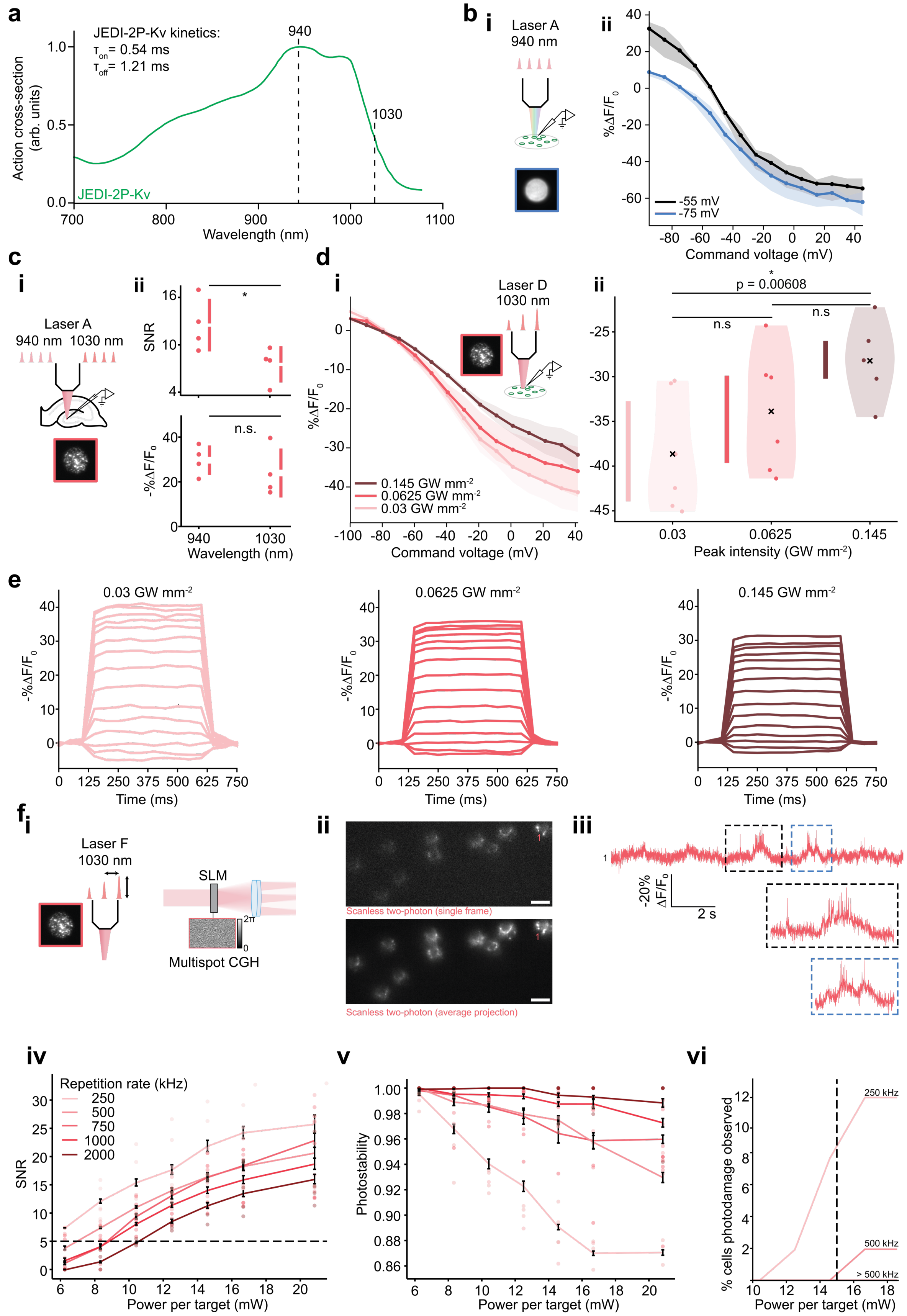

Supplementary Figure 10 - Comparison 940 vs 1030-nm illumination, evaluation of sensitivity curve for low repetition rate illumination and characterization of scanless 2P voltage imaging using different repetition rates

This figure accompanies Figure 5 of the main text.

(a) Fluorescence spectrum of the genetically encoded voltage indicator JEDI-2P-Kv (adapted from Liu et al.[8]). **(b)** (i) JEDI-2P-Kv expressing CHO cells were patched and illuminated with TF-GPC to measure the sensitivity curve of JEDI-2P-Kv at 940 nm. (ii) -%$\Delta F/F_0$ as a function of command voltage at a resting potential of – 55 mV (average resting potential of CHO cells, black) or -75 mV (average resting potential of neurons in the dentate gyrus of hippocampal organotypic slices, blue). The average trace and 95 percent confidence interval from all cells are plotted (n = 8 cells from 1 transfection). Data were acquired with laser A tuned at 940 nm, with power density: 0.88 mW μm^{-2} (100 mW per cell), 100 Hz acquisition rate and camera A (Refer to Supplementary Figure 1 and Supplementary Tables 1 and 4). **(c)** (i) JEDI-2P-Kv expressing neurons in hippocampal organotypic slices were patched and illuminated using CGH to compare the performances of the voltage indicator at 940 and 1030 nm. (ii) Comparison of SNR (upper) and -%$\Delta F/F_0$ (lower) of electrically evoked APs, between 940 and 1030 nm illumination (n = 4 neurons, from 2 different slices from 1 slice culture). Data was acquired using laser A tuned to 940 or 1030 nm, with same photon flux (6.29 x 10^{27} photons s^{-1} μm^{-2}), corresponding to power densities of 1.33 mW μm^{-2} at 940 nm and 1.21 mW μm^{-2} at 1030 nm (powers: 150 and 137 mW respectively). **(d)** (left) -%$\Delta F/F_0$ as a function of command voltage at a resting potential of -75 mV with different peak intensities. The average trace and 95 percent confidence interval from all cells are plotted (n = 6 from 1 transfection). (right) -%$\Delta F/F_0$ for a 100-mV depolarization step with different peak intensities. Each point represents data from an individual cell. All data were acquired at 1030 nm using laser D, with peak intensities: 0.03, 0.0625 and 0.145 GW/mm^{-2}, (20, 43 and 98 mW per cell), 5 Hz acquisition rate and camera A (Refer to Supplementary Figure 1 and Supplementary Tables 1, 2 and 4). * denotes $p<0.05$, ** denotes $p<0.01$ and *** denotes $p<0.0001$ (t-test, see Methods). **(e)** Average fluorescence responses recorded using different illumination intensities (as labelled). The mean of 3 cells is plotted. **(f)** Characterisation of scanless 2P voltage imaging of JEDI-2P-Kv with 1030 nm excitation as a function of repetition rate and average power. (i) JEDI-2P-Kv expressing neurons in hippocampal organotypic slices were illuminated simultaneously using TF-CGH. (ii) Single frame (upper) and average temporal projection (lower) of data acquired during a representative multi-target scanless 2P voltage imaging experiment (power density: 0.03 mW μm^{-2}, power: 7.5 mW per cell, acquisition rate 500 Hz, illumination duration: 30 s). (iii) Representative trace of spontaneous activity recorded from cell 1 (see label in (ii)). Inset: zoomed in portion of the APs recorded. (iv) SNR and (v) photostability plotted as a function of average power (6 - 21 mW, corresponding to power densities of 0.03 - 0.09 mW μm^{-2}) for different repetition rates between 250 – 2000 kHz (see legend) (n = 27 - 50 neurons per repetition rate, from 3 - 5 different slices from 1 slice culture). (vi) Percentage of cells where photodamage was observed plotted as a function of power per cell, for different laser repetition rates. The maximum power per target used in this manuscript (15 mW) using this laser is indicated by the black dashed line.

Supplementary Figure 11

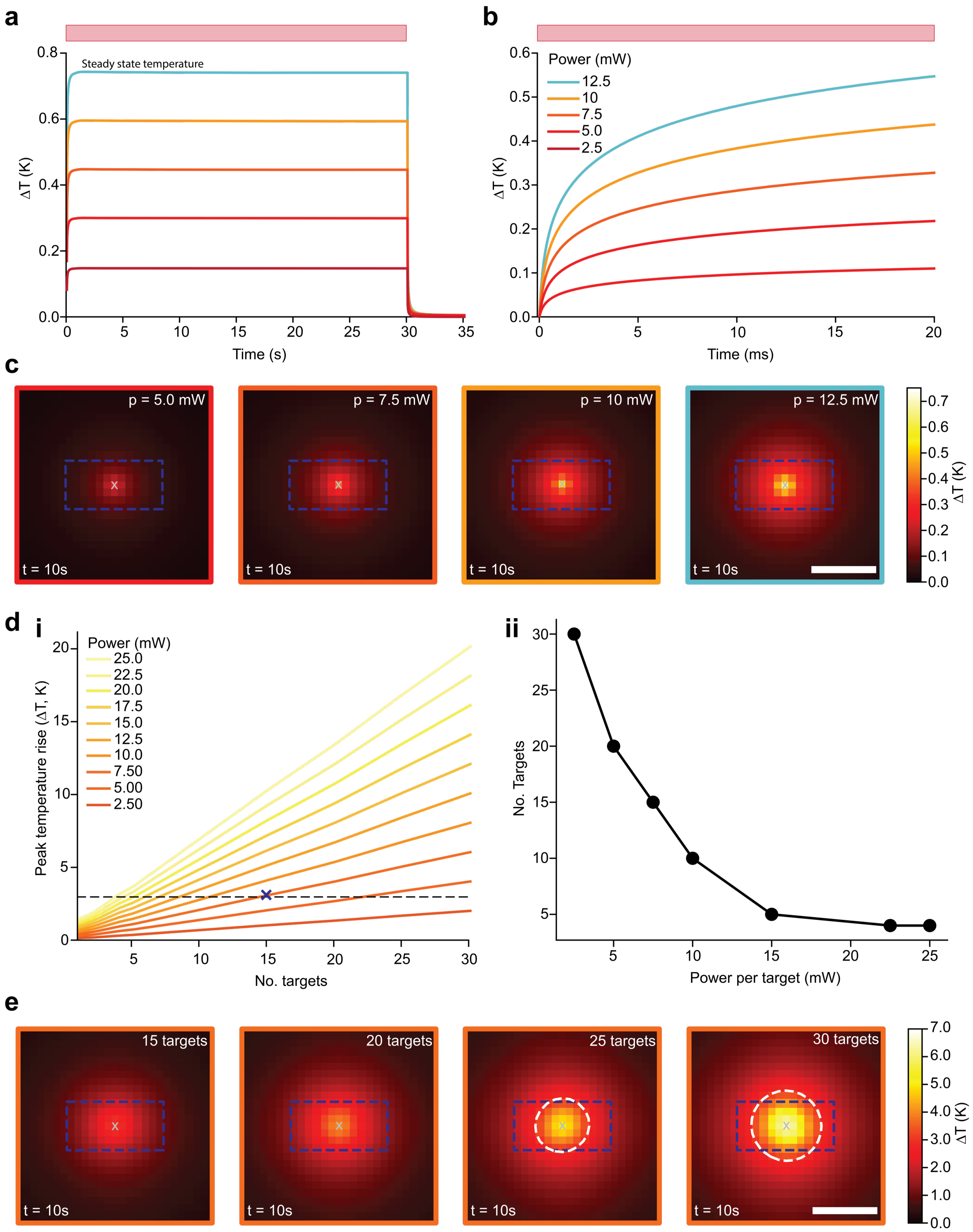

Supplementary Figure 11 – Simulations of temperature rises induced by scanless voltage imaging with low repetition rate laser pulses in vitro

This figure accompanies Figure 5 of the main text.

(**a-b**) Temperature rise as a function of time for different illumination powers used for scanless 2P voltage imaging of single neurons in superficial layers using a low repetition rate laser at 1030 nm. Results from different illumination powers are plotted in different colours (refer to the legend in b). (**c**)

Spatial profiles of the temperature rises induced by scanless 2P voltage imaging at different average illumination powers (indicated top right) after 10 s. The dashed blue boxes indicate the imaging field of view corresponding to a 500 Hz acquisition rate. In each case, the illuminated neuron (simulated) was located in the centre of the field of view. (**d**) (i) Simulated peak temperature rise (during a 30 s recording) as a function of the number of neurons imaged simultaneously using scanless 2P voltage imaging using different average power per cell (see legend). (ii) Number of targets that can be imaged simultaneously whilst maintaining the light induced temperature rise below 3 K as a function of the power per target cell. (**e**) Spatial profiles of the temperature rises induced by scanless 2P voltage imaging for different number of targets (indicated top right) after 10 s, using 7.5 mW average power per cell. The dashed blue boxes indicate the imaging field of view corresponding to a 500 Hz acquisition rate. In each case, the targeted neurons (simulated) were maximally distributed throughout the field of view (see Methods).

Supplementary Figure 12

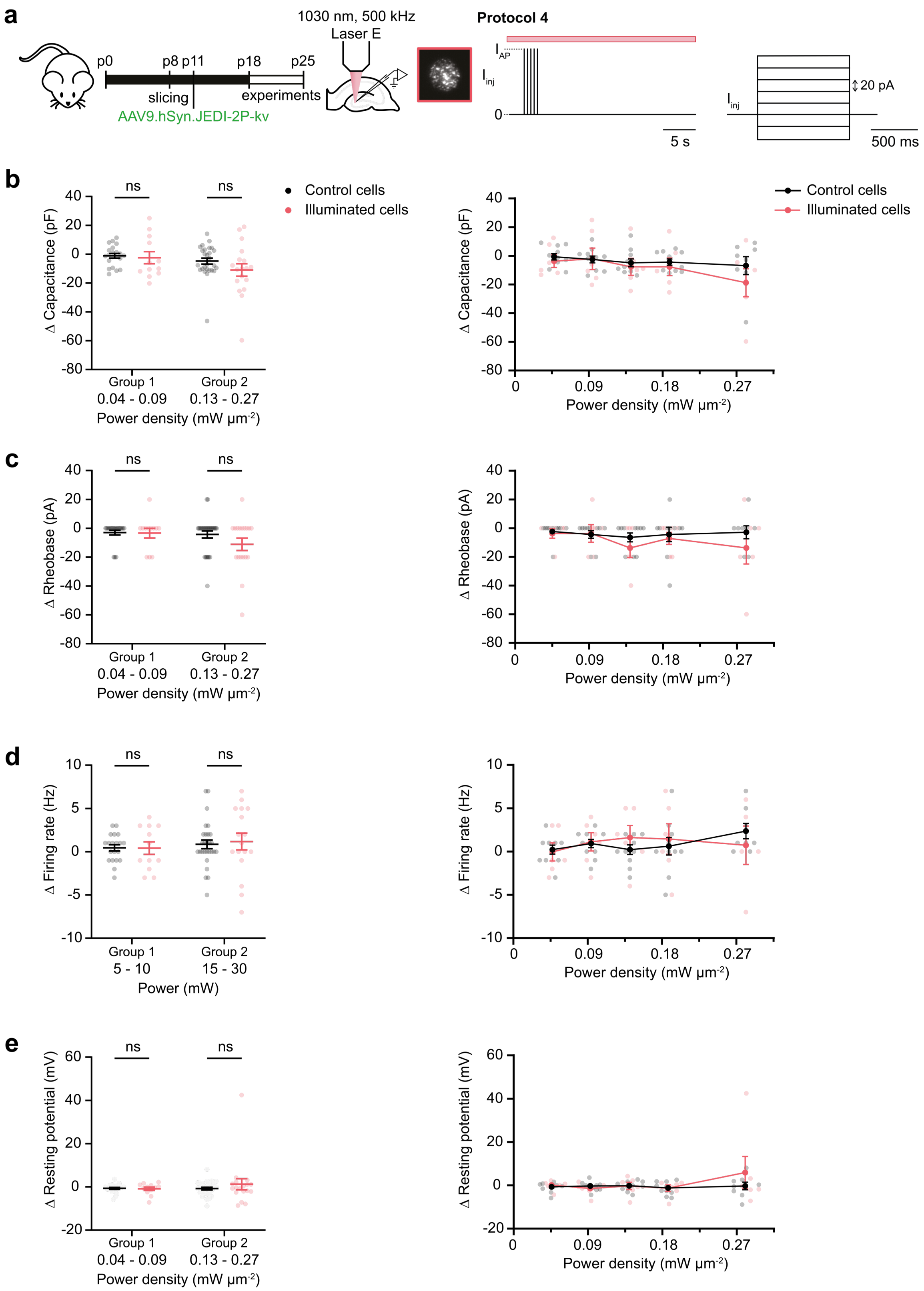

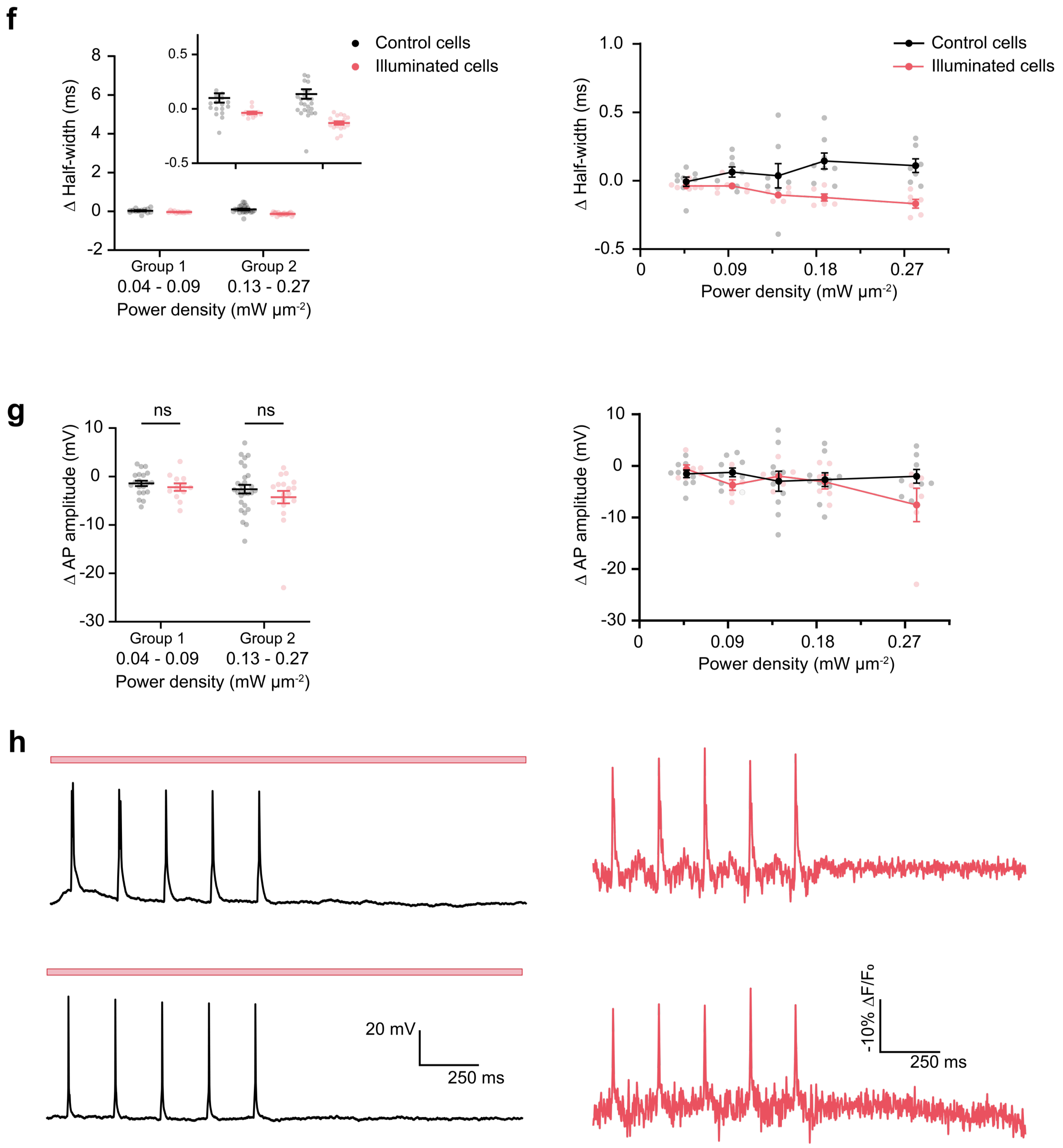

Supplementary Figure 12 - Quantification of perturbations induced by scanless 2P voltage imaging upon illumination with laser pulses at low repetition rate

This figure accompanies Figure 5 of the main text.

(a) Schematics of the experimental protocol (for more details, refer to Methods). **(b - g)** Relative changes in (b) capacitance, (c) rheobase, (d) firing rate, (e) resting potential, (f) AP half-width and (g) AP amplitude as measured using whole-cell patch clamp electrophysiology are plotted as a function of power (protocol 4, see Methods and Supplementary Table 5, red points). The black points are control cells which were patched but not illuminated. Each point represents data from an individual trial. (Left) Data from different cells and power densities have been pooled into two groups (group 1: 0.04 - 0.09 mW μm^{-2} and group 2: 0.13 - 0.27 mW μm^{-2}, corresponding to powers per cell of 5 - 10 mW and 15 - 30 mW respectively) (n = 5 - 6 cells, 2 independent transductions). The population average and standard error are indicated in black or red in each case. * denotes p<0.05, ** denotes p<0.01 and *** denotes p<0.0001 (Mann-Whitney *U*-test). (Right) Data from individual cells are plotted as a function of average incident power density (0.13 - 0.27 mW μm^{-2}, powers per cell: 5 - 30 mW) used for scanless 2P imaging (protocol 4, see Methods and Supplementary Table 5, red points). Each point represents data from a single trial. All data was acquired at 1030 nm excitation using laser E, 1 MHz repetition rate. **(h)** Representative electrophysiological (left) and corresponding fluorescence traces (right) of 5

electrically induced APs recorded at power densities of 0.66 mW μm^{-2} (power per cell: 75 mW), before (upper) and after (lower) 3 min of continuous illumination. Data acquired using laser D (1030 nm, 40 MHz repetition rate) and camera A (Refer to Supplementary Figure 1 and Supplementary Tables 1 and 2).

Supplementary Figure 13

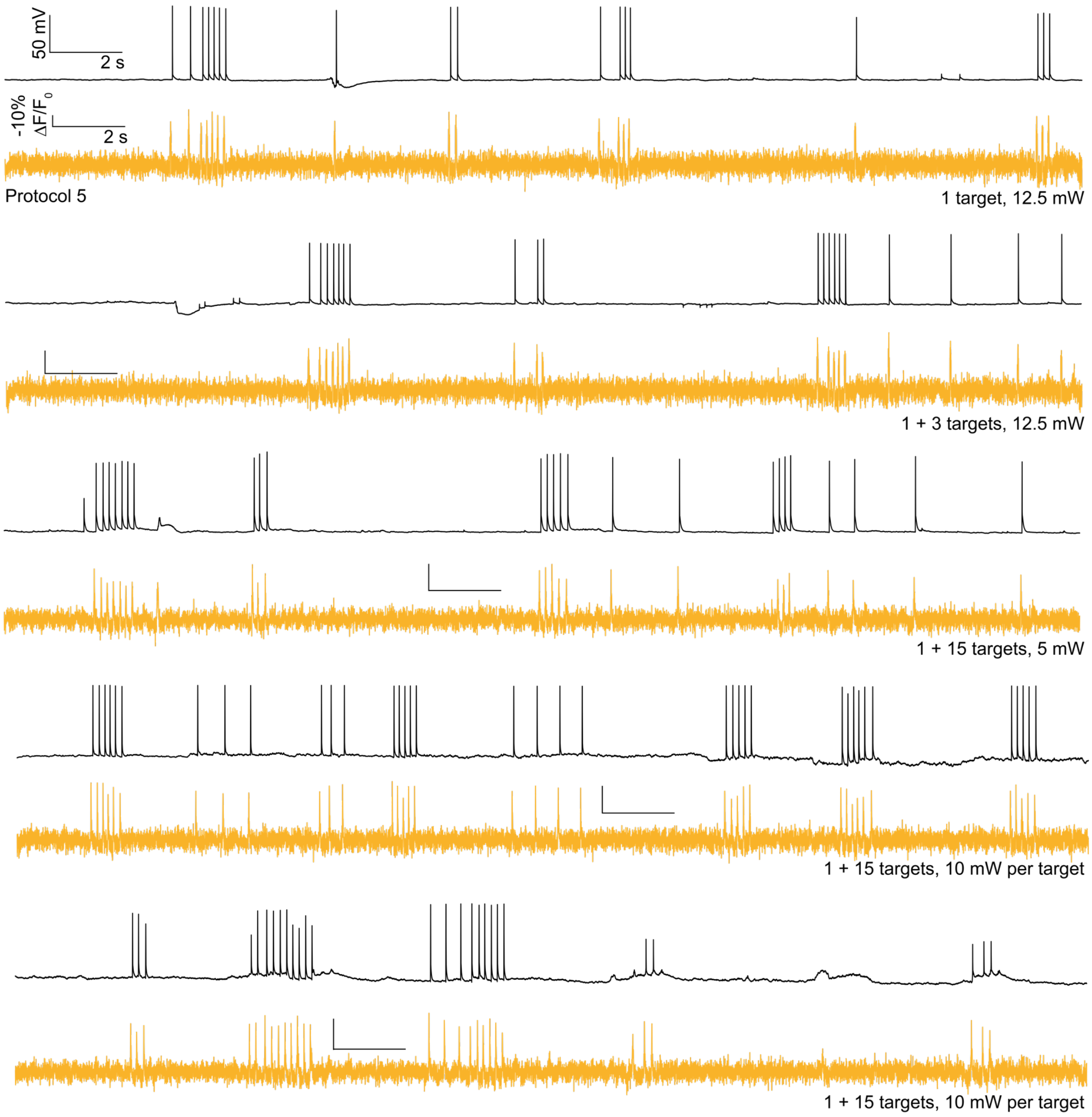

Supplementary Figure 13 - Multi-target scanless 2P voltage imaging with low repetition rate illumination at 1030 nm

This figure accompanies Figure 5 of the main text.

Examples of simultaneous current clamp (upper, black) and fluorescence recordings (lower, yellow) of electrically evoked activity in neurons from hippocampal organotypic slices (protocol 5, Methods). Data was acquired using TF-Gaussian in combination with a low-repetition rate (500 kHz) source at 1030 nm (Laser E). The duration of each recording was 30 s. Either 0, 3 or 15 additional spots (as specified in each figure inset) were randomly positioned in the densely labelled field of view. Only data from the spot directed towards the patched cell is plotted. Average powers between 5 – 12.5 mW per target were used (as indicated).

Supplementary Figure 14

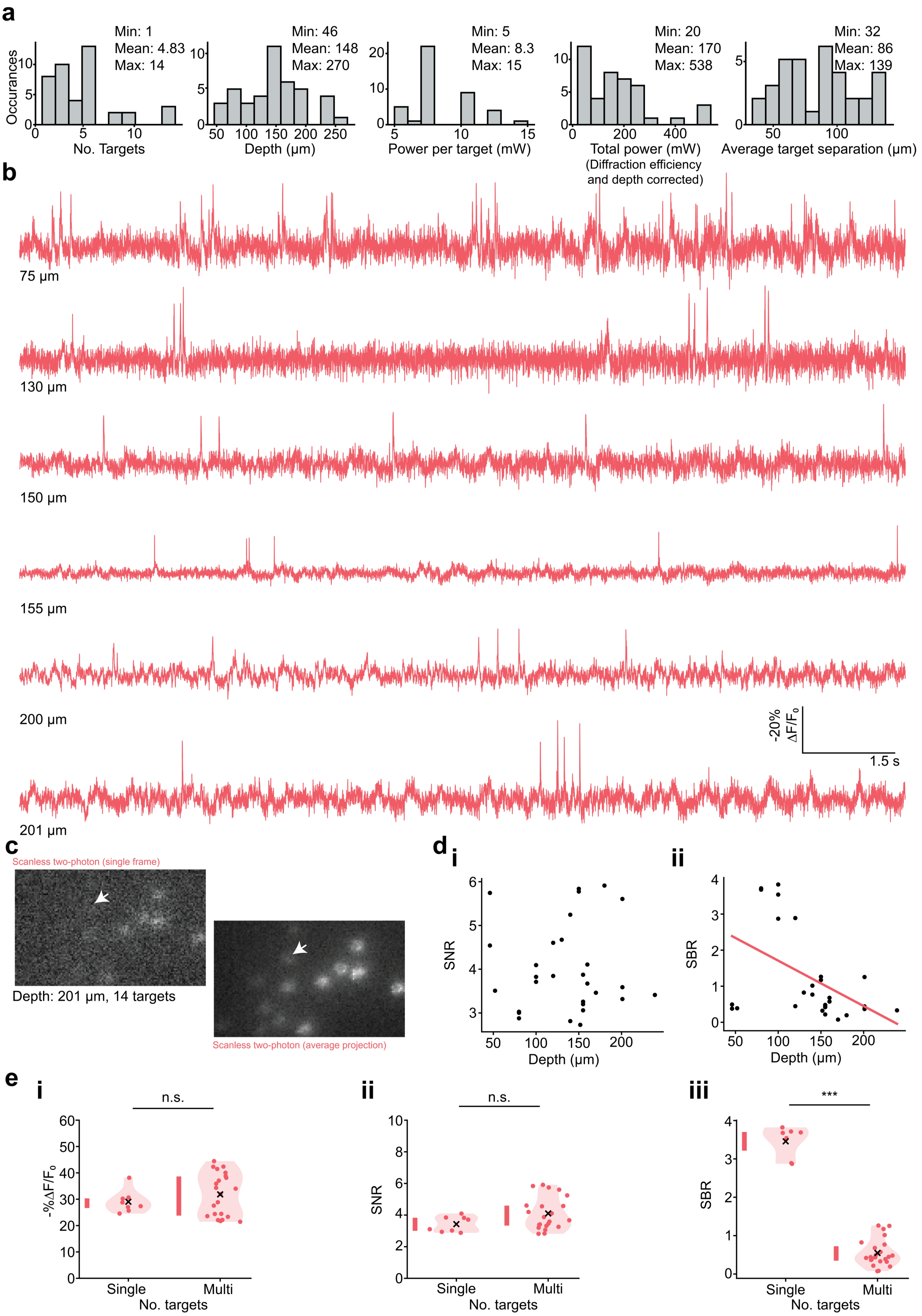

Supplementary Figure 14 – In vivo scanless 2P voltage imaging

This figure accompanies Figure 6 of the main text.

(**a**) Summary statistics for in vivo scanless 2P imaging experiments. The minimum, mean and maximum are indicated in each case. (i) Number of target neurons per acquisition, (ii) depth of imaging plane below cortical surface (μm), (iii) desired average power per target, (iv) total power delivered to sample (measured after objective), (v) average Euclidean separation between targets. (**b**) Traces from single neurons (acquired during multi-target experiments), at different depths (indicated) in the barrel cortex below the cortical surface. (**c**) Representative multi-cell imaging data acquired 201 μm below the cortical surface in vivo. 14 neurons were targeted simultaneously. The arrow indicates the neuron corresponding to the trace at 201 μm plotted in Figure 5 of the main text. (**d**) (i - ii) Signal to noise ratio (SNR) and signal to background ratio (SBR) plotted as a function of imaging depth below the cortical surface. That the SNR does not change as a function of depth is likely an artifact of the prototype system used to acquire data which biased deeper recordings to target the most highly expressing cells. (**e**) Characterisation of the -%$\Delta F/F_0$, SNR, and signal-to-background ratio (SBR), of fluorescence traces of spontaneous APs acquired with scanless 2P voltage imaging (TF-CGH) whilst targeting single or multiple (multi) cells. In each case each coloured point represents a single measurement from an individual cell, the black cross is located at the population mean and the coloured bars (adjacent) depict the interquartile range. * denotes $p<0.05$, ** denotes $p<0.01$ and *** denotes $p<0.0001$. All data was acquired using TF-CGH at 1030 nm, 500 kHz repetition rate (laser F), 30 s recordings, 500 Hz acquisition rate. Average powers per neuron 5 – 15 mW.

Supplementary Figure 15

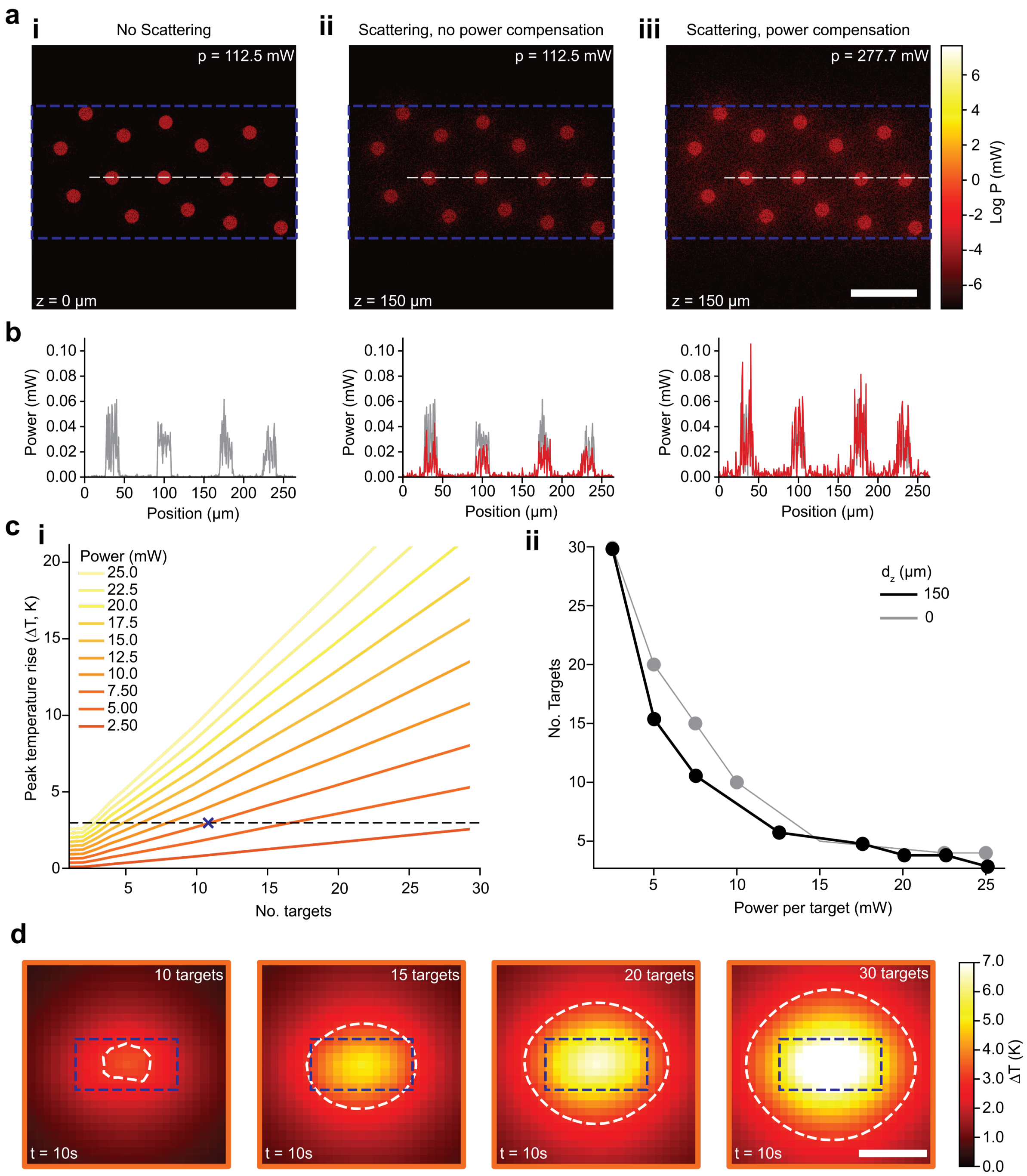

Supplementary Figure 15 - Simulations of temperature rises upon scanless voltage imaging of multiple neurons simultaneously 150 µm deep in scattering tissue

This figure accompanies Figure 6 of the main text.

(a) The light distribution of 15 multiplexed holographic spots at different depths and for different incident powers: (i) superficial cortical layers (no scattering) (ii) 150 µm deep in cortical tissue without compensating for the power loss in each target due to scattering (iii) 150 µm deep in cortical tissue compensating for the power loss in each target due to scattering. The data is presented on a log scale to better visualize the scattered light between targeted neurons (refer to colour bar). **(b)** Line profiles from the data in (a) corresponding to the white dashed line. The grey plot corresponds to the no-scattering case and is plotted in all three graphs. (**c**) (i) Temperature rise as a function of number of targets for different illumination powers used for scanless 2P voltage imaging of multiple neurons 150

μm below the cortical surface using a low repetition rate laser at 1030 nm. Results from different illumination powers are plotted in different colours (refer to the legend in c(i)). (ii) Number of targets that can be imaged simultaneously whilst maintaining the light induced temperature rise below 3 K as a function of the power per target cell. (**d)** Spatial profiles of the temperature rises induced by scanless 2P voltage imaging for different number of targets (indicated top right) after 10 s, using 7.5 mW average power per cell (compensated for imaging 150 μm below the cortical surface). The dashed blue boxes indicate the imaging field of view corresponding to a 500 Hz acquisition rate. The white dashed contours indicate a 3 K temperature rise. In each case, the targeted neurons (simulated) were maximally distributed throughout this field of view (see Methods).

Supplementary Figure 16

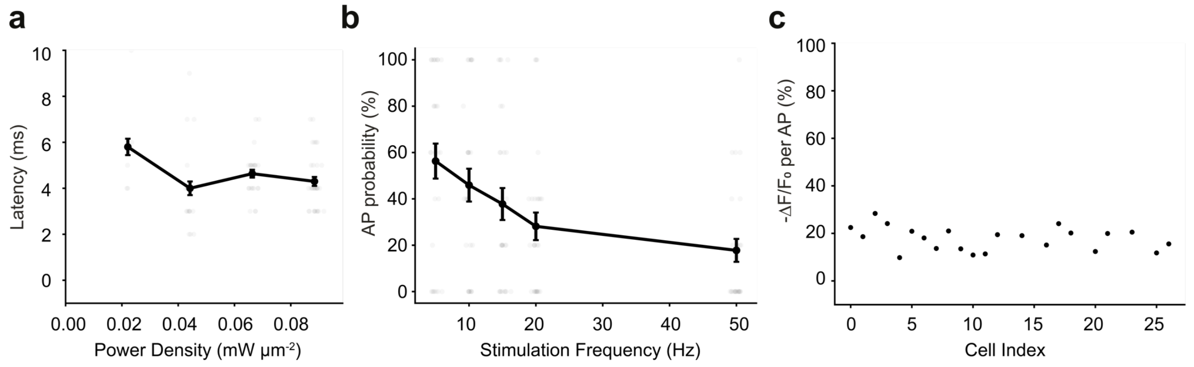

Supplementary Figure 16 - All-optical in situ characterization of photo evoked action potentials

This figure accompanies Figure 7 of the main text.

(a) Latency of optically evoked APs (defined as the time between the onset of stimulation and the peak of the action potential) plotted as a function of power density. The average latency measured all-optically matches that obtained using electrophysiology (data not shown). **(b)** AP probability plotted as a function of stimulation frequency. AP probability is calculated as the number of APs evoked and recorded over five trials (power density: 0.01 – 0.09 mW μm^{-2}, 1.5 – 10 mW per cell). Error bars represent the standard error of recordings obtained for 27 repetitions. **(c)** Average -%$\Delta F/F_0$ of optically evoked APs for 27 cells (power density: 0.02 – 0.08 mW μm^{-2}, 2.5 – 9 mW per cell) recorded at 500 Hz. All data were acquired using laser C fixed at 940 nm and camera A (See Supplementary Figure 1 and Supplementary Tables 1 and 4).

Supplementary Figure 17

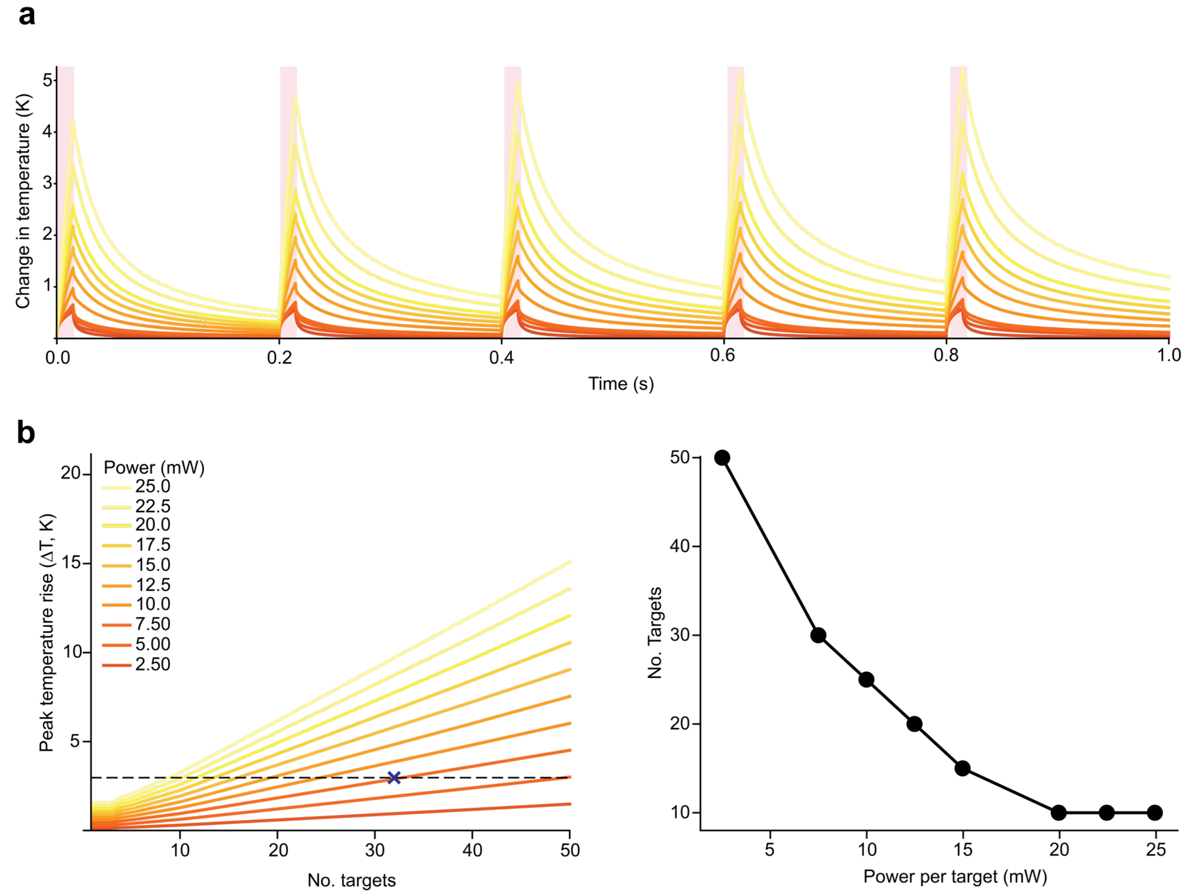

Supplementary Figure 17 – Simulation of temperature rises in tissue upon scanless voltage imaging with low duty cycles and single-beam photoactivation and voltage imaging

This figure accompanies Figure 7 of the main text.

(a) Temperature rise as a function of time for different illumination powers used for scanless 2P voltage imaging of single neurons in superficial layers using a low repetition rate laser at 1030 nm and a low duty cycle illumination protocol (5 Hz, 20 ms illumination). Illumination periods are indicated by the red boxes. **(b)** Left panel: Temperature rise (units: Kelvin) as a function of number of targets for different illumination powers used for scanless 2P voltage imaging of multiple neurons 150 μm below the cortical surface using a low repetition rate laser at 1030 nm and a low-duty cycle protocol (5 Hz, 20 ms illumination). Results from different illumination powers are plotted in different colours (refer to the legend in b). Right panel: Number of targets that can be imaged simultaneously whilst maintaining the light induced temperature rise below 3 K as a function of the power per target cell.

Supplementary References

1. Roome, C. J. & Kuhn, B. Simultaneous dendritic voltage and calcium imaging and somatic recording from Purkinje neurons in awake mice. *Nat Commun* **9**, 1–14 (2018).

2. Brinks, D., Klein, A. J. & Cohen, A. E. Two-Photon Lifetime Imaging of Voltage Indicating Proteins as a Probe of Absolute Membrane Voltage. *Biophys J* **109**, 914–921 (2015).

3. Fisher, J. a N. *et al.* Two-photon excitation of potentiometric probes enables optical recording of action potentials from mammalian nerve terminals in situ. *J Neurophysiol* **99**, 1545–1553 (2008).

4. Wu, J. *et al.* Kilohertz two-photon fluorescence microscopy imaging of neural activity in vivo. *Nat Methods* **17**, 287–290 (2020).

5. Villette, V. *et al.* Ultrafast Two-Photon Imaging of a High-Gain Voltage Indicator in Awake Behaving Mice. *Cell* **179**, 1590-1608.e23 (2019).

6. Chamberland, S. *et al.* Fast two-photon imaging of subcellular voltage dynamics in neuronal tissue with genetically encoded indicators. *Elife* **6**, 1–35 (2017).

7. Platisa, J. *et al.* High-speed low-light in vivo two-photon voltage imaging of large neuronal populations. *Nat Methods* (2023) doi:10.1038/s41592-023-01820-3.

8. Liu, Z. *et al.* Sustained deep-tissue voltage recording using a fast indicator evolved for two-photon microscopy. *Cell* **185**, 3408-3425.e29 (2022).

9. Evans, S. W. *et al.* A positively tuned voltage indicator for extended electrical recordings in the brain. *Nat Methods* **20**, 1104–1113 (2023).

10. Cai, C. *et al.* VolPy: Automated and scalable analysis pipelines for voltage imaging datasets. *PLoS Comput Biol* **17**, 1–27 (2021).

11. Abdelfattah, A. S. *et al.* Bright and photostable chemigenetic indicators for extended in vivo voltage imaging. *Science (1979)* **365**, 699–704 (2019).

12. Buchanan, E. K. *et al.* Penalized matrix decomposition for denoising, compression, and improved demixing of functional imaging data. 1–36 (2018).

13. Van Der Walt, S. *et al.* Scikit-image: Image processing in python. *PeerJ* **2014**, 1–18 (2014).
