# Supplementary material for: Scanless two-photon voltage imaging
Source: Nat Commun. 2024 Jun 14;15:5095. doi: 10.1038/s41467-024-49192-2 (PMC11178882; doi:10.1038/s41467-024-49192-2)
Supplement: Supplementary file 3 — Reporting Summary [file 41467_2024_49192_MOESM3_ESM.pdf]

nature portfolio

Corresponding author(s): Valentina Emiliani, Eirini Papagiakoumou

Last updated by author(s): Apr 29, 2024

Reporting Summary

Nature Portfolio wishes to improve the reproducibility of the work that we publish. This form provides structure for consistency and transparency in reporting. For further information on Nature Portfolio policies, see our Editorial Policies and the Editorial Policy Checklist.

Statistics

For all statistical analyses, confirm that the following items are present in the figure legend, table legend, main text, or Methods section.

n/a	Confirmed	
☐	☒	The exact sample size (*n*) for each experimental group/condition, given as a discrete number and unit of measurement
☐	☒	A statement on whether measurements were taken from distinct samples or whether the same sample was measured repeatedly
☐	☒	The statistical test(s) used AND whether they are one- or two-sided<br>*Only common tests should be described solely by name; describe more complex techniques in the Methods section.*
☐	☒	A description of all covariates tested
☐	☒	A description of any assumptions or corrections, such as tests of normality and adjustment for multiple comparisons
☐	☒	A full description of the statistical parameters including central tendency (e.g. means) or other basic estimates (e.g. regression coefficient) AND variation (e.g. standard deviation) or associated estimates of uncertainty (e.g. confidence intervals)
☐	☒	For null hypothesis testing, the test statistic (e.g. *F*, *t*, *r*) with confidence intervals, effect sizes, degrees of freedom and *P* value noted<br>*Give P values as exact values whenever suitable.*
☒	☐	For Bayesian analysis, information on the choice of priors and Markov chain Monte Carlo settings
☒	☐	For hierarchical and complex designs, identification of the appropriate level for tests and full reporting of outcomes
☒	☐	Estimates of effect sizes (e.g. Cohen's *d*, Pearson's *r*), indicating how they were calculated

Our web collection on statistics for biologists contains articles on many of the points above.

Software and code

Policy information about availability of computer code

Data collection	Python 3.8.15, Micro-Manager 2.0.0, pClamp 11
Data analysis	Python 3.8.15, Graphpad Prism 9.0, pClamp 11

For manuscripts utilizing custom algorithms or software that are central to the research but not yet described in published literature, software must be made available to editors and reviewers. We strongly encourage code deposition in a community repository (e.g. GitHub). See the Nature Portfolio guidelines for submitting code & software for further information.

Data

Policy information about availability of data

All manuscripts must include a data availability statement. This statement should provide the following information, where applicable:

- Accession codes, unique identifiers, or web links for publicly available datasets
- A description of any restrictions on data availability
- For clinical datasets or third party data, please ensure that the statement adheres to our policy

Due to the quantity of data acquired in total, the data that support the findings of this study are available from the corresponding author on reasonable request. Source data will be provided with the manuscript along with representative datasets.

Research involving human participants, their data, or biological material

Policy information about studies with human participants or human data. See also policy information about sex, gender (identity/presentation), and sexual orientation and race, ethnicity and racism.

Reporting on sex and gender	N/A
Reporting on race, ethnicity, or other socially relevant groupings	N/A
Population characteristics	N/A
Recruitment	N/A
Ethics oversight	N/A

Note that full information on the approval of the study protocol must also be provided in the manuscript.

Field-specific reporting

Please select the one below that is the best fit for your research. If you are not sure, read the appropriate sections before making your selection.

☒ Life sciences ☐ Behavioural & social sciences ☐ Ecological, evolutionary & environmental sciences

For a reference copy of the document with all sections, see nature.com/documents/nr-reporting-summary-flat.pdf

Life sciences study design

All studies must disclose on these points even when the disclosure is negative.

Sample size	Sample-size calculations were not performed as the effect size was not known before the study. However, in each case we aimed to exceed (mostly doubling) typical sample sizes used to perform comparable experiments for validating novel voltage imaging approaches (for instance: Villette et al, Cell, 2019; Liu et al, Cell, 2022; Platisa et al, Nature Methods, 2023).
Data exclusions	For patch-clamp experiments, recordings were excluded if the recording was not sufficiently stable throughout the duration of the experiment or if the quality of the patch was insufficient to clamp the membrane potential to the necessary voltage with sufficient accuracy. In all cases, only recordings with an access resistance below 35 MOhm were included in subsequent analysis. For current-clamp recordings performed in hippocampal organotypic slices, only cells which were maintained at the original (break-in) resting membrane potential via current injection smaller than 100 pA were included in subsequent analysis.
Replication	All experiments were repeated multiple times from multiple preparations (CHO cells, hippocampal organotypic slices, mice) and multiple (at least two) transfections/ infections/ injections in each case, as reported in the Methods section of the manuscript. These experiments had comparable outcomes in all cases.
Randomization	In general, it was not necessary to allocate data into different experimental groups. There were two main instances where this was necessary. Firstly for the CHO cell experiments, different measurements performed on single cells (for instance using different powers) were performed in a random order (random numbers generated using Python). The second instance was when measuring the effect of scanless voltage imaging on cell health using organotypic slices. In this case it was necessary to perform the experiments in the same order to identify the onset of damage. In this case, measurements were performed in the same order when acquiring both the control and non-control datasets.<br><br>In general, target cells were randomly selected from those that appeared to be in good health (as determined from transmitted light images), well adhered to the cover slip and were fluorescent. Hippocampal slice cultures were randomly chosen for virus transduction. For the in-vivo experiments, all cells in the field of view were selected and targeted in consecutive acquisitions.
Blinding	Blinding was not possible during data acquisition because it was necessary to identify which cells were fluorescent and also to change the experimental configuration to use a particular excitation modality.<br><br>Data analysis was not performed blindly since the modality used for excitation was apparent in the raw images. However, an automated data analysis pipeline was established by pooling data acquired using all modalities and then used to analyse all data acquired using a particular protocol (without changing any parameters) , as outlined in the manuscript.

Reporting for specific materials, systems and methods

We require information from authors about some types of materials, experimental systems and methods used in many studies. Here, indicate whether each material, system or method listed is relevant to your study. If you are not sure if a list item applies to your research, read the appropriate section before selecting a response.

Materials & experimental systems

n/a	Involved in the study	
☒	☐	Antibodies
☐	☒	Eukaryotic cell lines
☒	☐	Palaeontology and archaeology
☐	☒	Animals and other organisms
☒	☐	Clinical data
☒	☐	Dual use research of concern
☒	☐	Plants

Methods

n/a	Involved in the study	
☒	☐	ChIP-seq
☒	☐	Flow cytometry
☒	☐	MRI-based neuroimaging

Eukaryotic cell lines

Policy information about cell lines and Sex and Gender in Research

Cell line source(s)	CHO cells (ECACC 85050302, purchased from Sigma-Aldrich, distributor in Europe for ECACC)
Authentication	CHO cells were authenticated by ECACC before purchase and only passaged until P20 to avoid genetic drift
Mycoplasma contamination	Cell lines were not tested for mycoplasma contamination
Commonly misidentified lines (See ICLAC register)	No commonly misidentified cell lines were used in this study

Animals and other research organisms

Policy information about studies involving animals; ARRIVE guidelines recommended for reporting animal research, and Sex and Gender in Research

Laboratory animals	Hippocampal organotypic slices were prepared from mice of both sexes (C57Bl6J, purchased from Janvier Labs), at postnatal day 8 (P8). In vivo experiments were performed on adult mice of both sexes (week 7 - 17) (C57Bl6J, Janvier Labs)
Wild animals	No wild animals were used in this study
Reporting on sex	Animals of both sexes were used for experiments
Field-collected samples	No field-collected samples were used in this study
Ethics oversight	Male and female C57BL/6J mice (Janvier Labs) were used for experiments, which were performed in accordance with EU Directive 2010/63. Protocols were reviewed by the local Animal Experimentation Ethics Committee (CETEA n.44) and approved by the French Ministry of Research and Education (#201803261541580). Advice on procedures, refinement of animal experimentation standards, and pain and distress management were provided by the Local Animal Welfare Office.

Note that full information on the approval of the study protocol must also be provided in the manuscript.

Plants

Seed stocks	N/A
Novel plant genotypes	N/A
Authentication	N/A
